# Signaling Pathways Mediating the Cytotoxicity of (E)-4-(1-Epoxy-7,8-propenyl)phenyl Benzoate, a Phenylpropanoid from *Croton velutinus*, in MCF‑7 Cells

**DOI:** 10.1021/acsomega.6c04220

**Published:** 2026-10-02

**Authors:** Renata A. de Abrantes, Sâmia S. Duarte, Rafael C. Ferreira, Ana Paula G. M. Farias, Geraldo M. W. Amorim, Paulo B. A. Loureiro, Ramon R. M. de Souza, Valgrícia M. de Sousa, Gabriela B. de Lyra, Isabela C. F. Silva, Lucas S. Abreu, Eudes da S. Velozo, Juan C. R. Gonçalves, Marcelo S. da Silva, Josean F. Tavares, Marianna V. Sobral

**Affiliations:** † Postgraduate Program in Natural Products and Bioactive Synthetics, 28097 Federal University of Paraíba (UFPB), João Pessoa, Paraíba 58051-970, Brazil; ‡ Department of Organic Chemistry, Fluminense Federal University, Niterói, Rio de Janeiro 24020-141, Brazil; § Research Laboratory in Materia Medica, School of Pharmacy, Federal University of Bahia, Salvador, Bahia 40170-115, Brazil

## Abstract

Natural products represent an important source of candidates for breast cancer therapy. Here, we investigated the antitumor activity of (E)-4-(1-epoxy-7,8-propen)­phenylbenzoate (CV2), a phenylpropanoid isolated from *Croton velutinus* roots, in MCF-7 breast cancer cells and evaluated its toxicity profile in nontumor cells and zebrafish embryos. CV2 exhibited potent cytotoxic activity against MCF-7 cells (IC_50_= 4.63 ± 0.16 μM) and showed selectivity relative to nontumorigenic MCF-10A cells (SI = 9.69). CV2 also induced apoptosis, increased intracellular ROS levels, and modulated MAPK, NF-κB, and PKB/AKT signaling pathways. Pretreatment with N-acetylcysteine or MAPK inhibitors significantly attenuated CV2-induced cytotoxicity, supporting the involvement of oxidative stress and MAPK signaling in its biological effects. In toxicity studies, CV2 exhibited cytotoxicity toward peripheral blood mononuclear cells (PBMCs; IC_50_ = 1.75 ± 0.06 μM) and an LC_50_ of 3.07 μM in zebrafish embryos, accompanied by alterations in oxidative stress-related enzymes. Collectively, these findings demonstrate that CV2 possesses significant antitumor activity against breast cancer cells while highlighting the need for further studies to better define its safety profile and therapeutic potential.

## Graphical abstract

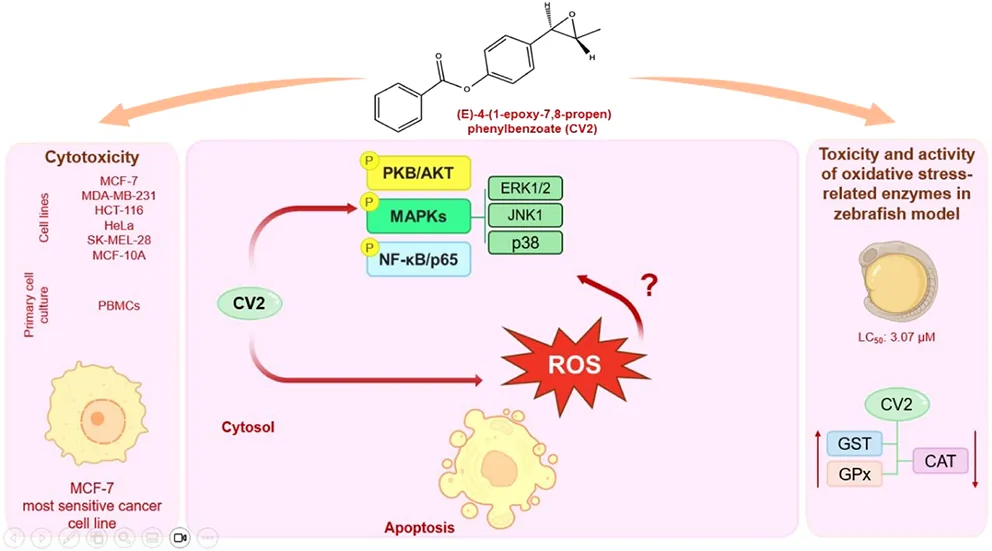

## 1 Introduction

Breast cancer arises from the uncontrolled proliferation of malignant cells within the mammary epithelium.^1^ According to the most recent GLOBOCAN estimates, approximately 2.3 million new breast cancer cases and 670,000 deaths occurred worldwide in 2022, making breast cancer the most frequently diagnosed cancer among women globally.^2^ The Global Burden of Disease Study 2023, published in 2026, reported 2.30 million incident cases and 764,000 deaths in 2023 and forecasts a substantial increase in the global burden, reaching 3.56 million new cases and 1.37 million deaths annually by 2050.^3^

Current treatment strategies for breast cancer include surgery, radiotherapy, chemotherapy, endocrine therapy, targeted therapies, and, for selected patient populations, immunotherapy. These approaches have significantly improved patient outcomes; however, treatment-related toxicity, disease recurrence, metastatic progression, and the development of therapeutic resistance remain major clinical challenges. Consequently, the identification of novel anticancer agents with improved efficacy and safety profiles continues to be an important objective in breast cancer research.^4^

Natural products and their phytochemicals are promising candidates for breast cancer prevention and treatment due to their structural diversity and ability to modulate multiple pathways involved in tumor progression.^5,6^ Among these compounds, phenylpropanoids have attracted considerable attention because of their antitumor potential and represent a promising source of novel anticancer agents.^7^

Recently, (E)-4-(1-epoxy-7,8-propen)­phenyl benzoate (CV2) (Figure 1), a phenylpropanoid isolated from the roots of *Croton velutinus* (Euphorbiaceae), showed significant cytotoxic activity against MCF-7 breast cancer cells in a preliminary study.^8^ Additionally, molecular docking analyses revealed favorable interactions between CV2 and key mitogen-activated protein kinases (MAPKs), including Extracellular signal-regulated kinases 1/2 (ERK1/2), c-Jun N-terminal kinase (JNK), and p38 mitogen-activated protein kinase (p38 MAPK), suggesting that these signaling proteins may contribute to its antitumor activity.^9^ However, the cellular and molecular mechanisms underlying the cytotoxic effects of CV2 remain poorly understood.

**Figure 1:**
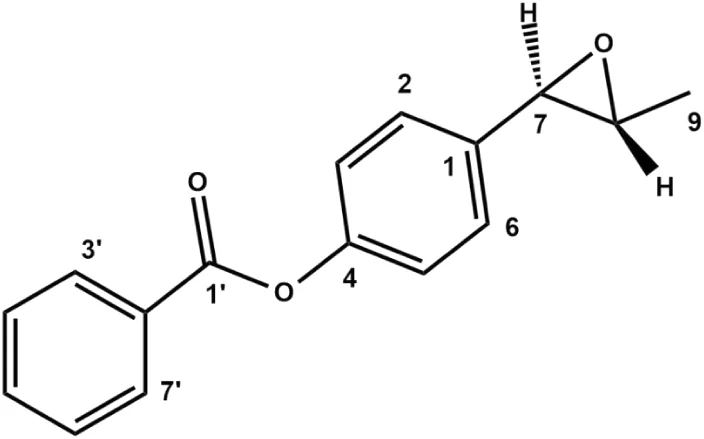
Molecular structure of (E)-4-(1-epoxy-7,8-propen)­fenilbenzoate (CV2).

Furthermore, several natural products induce antitumor responses through the modulation of key signaling pathways that regulate cancer cell proliferation, survival, stress responses, and apoptosis, including MAPKs, Protein Kinase B (PKB/AKT), and Nuclear Factor kappa B (NF-κB).^5,10,11^

MAPKs, particularly ERK1/2, JNK, and p38 MAPK, regulate fundamental cellular processes, including proliferation, differentiation, survival, apoptosis, and responses to extracellular stimuli.^12^ In contrast, PKB/AKT and NF-κB are more commonly associated with signaling pathways that promote cancer cell survival, proliferation, and therapeutic resistance.^13–15^ However, the biological effects of these signaling networks are highly context-dependent and may vary according to cellular conditions and downstream signaling events. In many cases, distinct patterns of activation have been associated with either tumor-promoting or tumor-suppressive responses, depending on the cellular context and the nature of the stimulus.^16–22^ Because dysregulation of these pathways is frequently observed in breast cancer, they represent relevant molecular targets for the investigation of novel anticancer agentes.

Importantly, MAPKs, PKB/AKT, and NF-κB are closely interconnected with cellular redox homeostasis and can be modulated by Reactive Oxygen Species (ROS). Although basal ROS levels contribute to tumor cell proliferation and survival, excessive ROS accumulation may activate stress-responsive signaling networks and trigger apoptotic cell death. Notably, ROS can modulate the activity of MAPKs, PKB/AKT, and NF-κB, while these pathways can, in turn, influence intracellular ROS production and antioxidant responses, establishing a complex regulatory crosstalk.^23–27^ Consistent with this interplay, several natural products have been reported to induce antitumor effects through ROS-dependent modulation of these signaling pathways.^10,28–31^ Accordingly, increasing evidence suggests that the modulation of ROS-dependent signaling pathways by natural products represents a promising therapeutic strategy for breast cancer, as these compounds can simultaneously target multiple molecular networks involved in tumor cell survival, proliferation, and apoptosis.^32^

Given the promising cytotoxic activity previously reported for CV2 and its predicted interactions with MAPK proteins, we hypothesized that this phenylpropanoid exerts antitumor effects through the modulation of redox-sensitive signaling pathways involved in cell survival and apoptosis. Therefore, the present study aimed to investigate the cytotoxic and toxicological profiles of CV2 in human tumor and nontumor cells, as well as in zebrafish embryos. In addition, we evaluated its effects on cell cycle progression, apoptosis induction, intracellular ROS production, and the modulation of ERK1/2, JNK, p38 MAPK, NF-κB (p65), and PKB/AKT signaling pathways in MCF-7 breast cancer cells. Collectively, these analyses were designed to provide new insights into the cellular and molecular mechanisms underlying the antitumor activity of CV2.

## 2 Results

### 2.1 Assessment of CV2 Cytotoxicity

#### 2.1.1 CV2 Induces Cytotoxicity in Human Cell Lines

CV2 exhibited cytotoxic activity against all tumor cell lines evaluated after 72 h of treatment (Table 1). Among them, MCF-7 breast cancer cells were the most sensitive to the compound (IC_50_ = 4.63 ± 0.16 μM), whereas HeLa cervical cancer cells were the least sensitive (IC_50_ = 51.78 ± 0.79 μM). In the nontumorigenic breast epithelial cell line MCF-10A, CV2 displayed lower cytotoxicity (IC_50_ = 44.09 ± 1.00 μM) than the standard drug doxorubicin (DXR; IC_50_ = 0.68 ± 0.07 μM).

**Table 1:** Cytotoxicity of (E)-4-(1-Epoxy-7,8-propen)­phenyl Benzoate (CV2) or Doxorubicin (DXR) against Human Tumor and Nontumor Cell Lines After 72 h of Treatment

	IC_50_ ^b^		SI^c^	
Cell lines^a^	CV2 (μM)	DXR (μM)	CV2	DXR
MCF-7	4.63 ± 0.16	1.53 ± 0.20	9.69	0.44
MDA-MB-231	23.72 ± 1.76	0.2 ± 0.02	1.85	3.4
HCT-116	12.85 ± 1.16	2.57 ± 0.01	3.43	0.26
HeLa	51.78 ± 0.79	3.80 ± 1.10	0.86	0.17
SK-MEL-28	16.62 ± 1.22	3.55 ± 1.67	2.65	0.19
MCF-10A	44.09 ± 1.00	0.68 ± 0.07	-	-

a MCF-7: human breast cancer; MDA-MB-231: triple-negative human breast cancer; HCT-116: human colon carcinoma; HeLa: human cervical cancer; SK-MEL-28: human melanoma; MCF-10A: nontumorigenic human breast epithelial cell line.

b IC_50_: Concentration that reduces 50% of cell viability.

c SI: Selectivity Index (IC_50_ nontumor cell line/IC_50_ tumor cell line). The data are expressed as mean ± standard error of the mean (SEM) from three independent experiments performed in triplicate (*n* = 3). IC_50_ values were estimated by nonlinear regression using a four-parameter logistic model based on log-transformed concentration–response curves.

The selectivity index (SI) was calculated using MCF-10A cells as the nontumor reference. As shown in Table 1, CV2 exhibited the highest selectivity toward MCF-7 cells (SI = 9.69), whereas DXR showed low selectivity for this cell line (SI = 0.44).

Based on its high sensitivity and selectivity, MCF-7 cells were selected for subsequent mechanistic studies. Time-course experiments revealed a progressive increase in the cytotoxic effect of CV2, with IC_50_ values decreasing from 41.29 ± 3.39 μM at 24 h to 29.22 ± 1.65 μM at 48 h and 4.63 ± 0.16 μM at 72 h (Figure 2).

**Figure 2:**
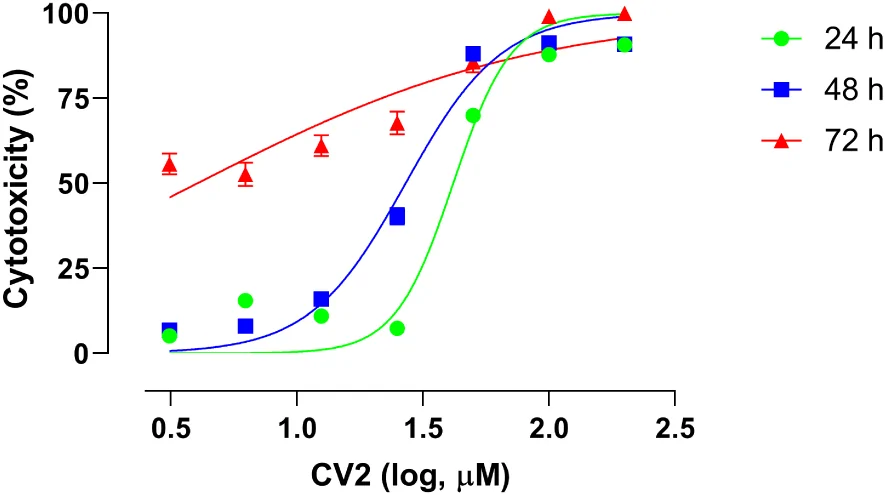
Concentration–response curves for the cytotoxic effects of (E)-4-(1-epoxy-7,8-propen)­phenyl benzoate (CV2) on MCF-7 human breast adenocarcinoma cells after 24, 48, and 72 h of treatment, as determined by the MTT assay. Data are presented as mean ± standard error of the mean (SEM) from three independent experiments performed in triplicate (*n* = 3). Concentrations are displayed on a logarithmic scale, and curves were fitted by nonlinear regression using a four-parameter logistic model for IC_50_ determination.

#### 2.1.2 CV2 Exhibits Lower Cytotoxicity toward PBMCs Than Doxorubicin

The cytotoxic effect of CV2 was also evaluated in human peripheral blood mononuclear cells (PBMCs). After 72 h of treatment, CV2 exhibited an IC_50_ value of 1.75 ± 0.06 μM, whereas doxorubicin (DXR) showed markedly higher cytotoxicity, with an IC_50_ value of 0.06 ± 0.006 μM (Figure 3).

**Figure 3:**
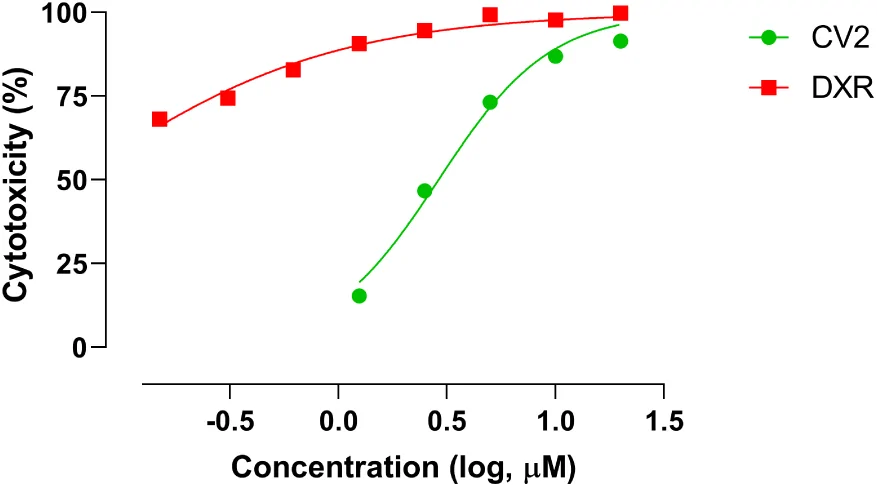
Concentration–response curves for the cytotoxic effects of (E)-4-(1-epoxy-7,8-propen)­phenyl benzoate (CV2) and doxorubicin (DXR) on human peripheral blood mononuclear cells (PBMCs) after 72 h of treatment, as determined by the MTT assay. Data are presented as mean ± standard error of the mean (SEM) from three independent experiments performed in triplicate (*n* = 3). Concentrations are displayed on a logarithmic scale, and curves were fitted by nonlinear regression using a four-parameter logistic model for IC_50_ determination.

### 2.2 CV2 Increases the Sub-G1 Cell Population

Treatment with CV2 altered cell cycle distribution in MCF-7 cells after 48 h of exposure (Figure 4). Both concentrations significantly increased the percentage of cells in the sub-G1 population (5 μM: 37.02 ± 0.25% and 10 μM: 11.29 ± 0.12%; *p* < 0.05 for both) compared with the control group (3.66 ± 0.10%). This increase was accompanied by a significant reduction in the proportion of cells in the G0/G1, S, and G2/M phases relative to the corresponding control group (*p* < 0.05 for all).

**Figure 4:**
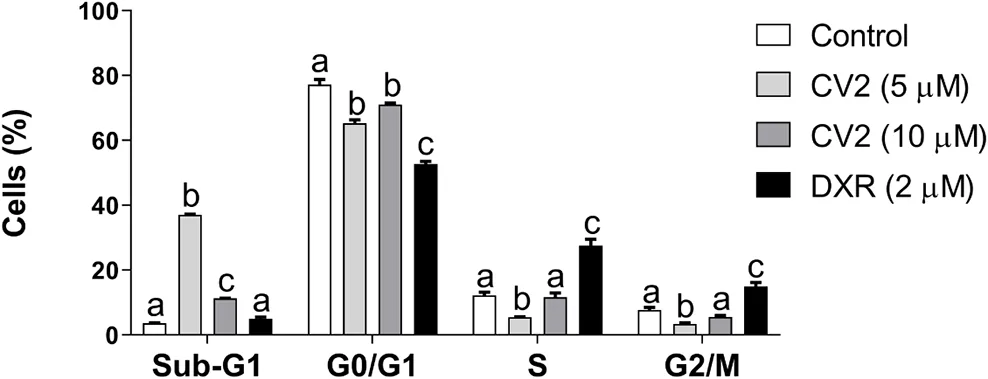
Effect of (E)-4-(1-epoxy-7,8-propen)­phenyl benzoate (CV2; 5 and 10 μM) and doxorubicin (DXR; 2 μM) on cell cycle distribution and sub-G1 population in MCF-7 human breast adenocarcinoma cells after 48 h of treatment. Data are presented as mean ± standard error of the mean (SEM) from three independent experiments performed in triplicate (*n* = 3). Statistical analysis was performed using one-way analysis of variance (ANOVA) followed by Tukey’s post hoc test. Different letters indicate statistically significant differences among groups within each cell cycle phase (*p* < 0.05), whereas groups sharing at least one letter do not differ significantly.

In contrast, doxorubicin (2 μM) induced a distinct cell cycle profile, characterized by a significant accumulation of cells in the S (27.52 ± 2.04%) and G2/M (14.96 ± 1.27%) phases compared with the control group (*p* < 0.05), indicating cell cycle arrest at these stages (Figure 4).

### 2.3 CV2 Induces Apoptosis in MCF-7 Cells

As shown in Figure 5, treatment with CV2 significantly increased the percentage of cells undergoing early apoptosis (Annexin V+/PI−) and the proportion of double-positive cells (Annexin V+/PI+), compared with the control group. Early apoptotic cells increased from 0.57 ± 0.10% in the control group to 27.78 ± 2.17% and 28.67 ± 1.19% following treatment with 5 and 10 μM CV2, respectively (*p* < 0.05 for both). Likewise, the percentage of Annexin V+/PI+ cells, indicative of late apoptosis and/or secondary necrosis increased from 3.56 ± 0.69% in the control group to 22.38 ± 1.32% and 15.83 ± 1.72% after treatment with 5 and 10 μM CV2, respectively (*p* < 0.05 for both). Consequently, the total proportion of Annexin V-positive cells (Annexin V+/PI– and Annexin V+/PI+) increased significantly at both concentrations, reaching 50.17 ± 2.52% and 44.50 ± 2.37% for 5 and 10 μM CV2, respectively, compared with 4.17 ± 0.32% in the control group (*p* < 0.05).

**Figure 5:**
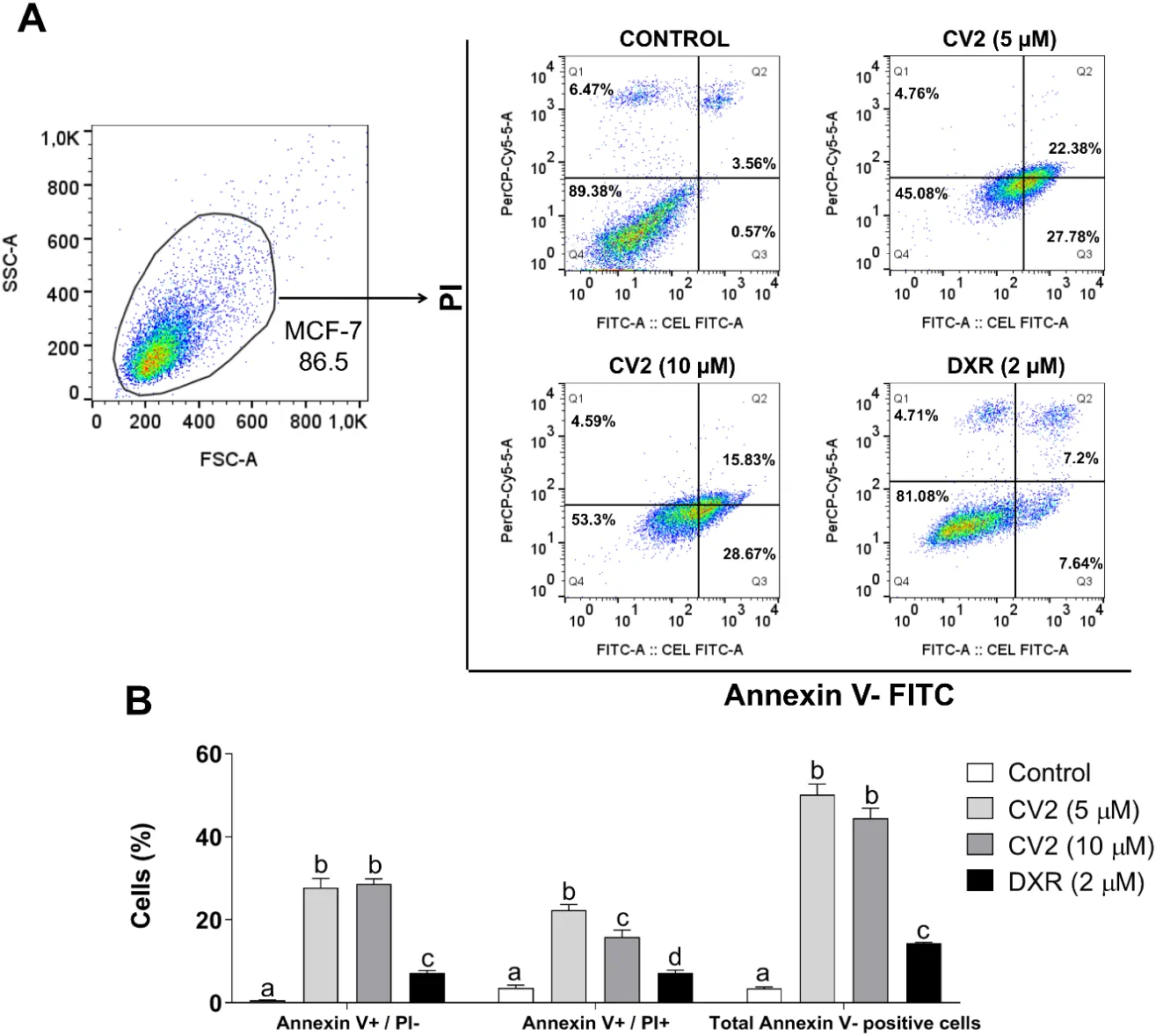
Effect of (E)-4-(1-epoxy-7,8-propen)­phenyl benzoate (CV2; 5 and 10 μM) and doxorubicin (DXR; 2 μM) treatment for 48 h on apoptosis induction in MCF-7 human breast adenocarcinoma cells, assessed by Annexin V–fluorescein isothiocyanate (FITC) and propidium iodide (PI) staining. (A) Representative flow cytometry dot plots are shown together with quantitative analysis of viable cells (Annexin V–/PI–, lower left quadrant), early apoptotic cells (annexin V+/PI–, lower right quadrant), late apoptotic cells (annexin V+/PI+, upper right quadrant), and necrotic cells (annexin V–/PI+, upper left quadrant). (B) Percentage of cells labeled with annexin-V-FITC and/or PI. Total Annexin V-positive cells correspond to the combined Annexin V+/PI– and Annexin V+/PI+ populations. Data are presented as mean ± standard error of the mean (SEM), from three independent experiments performed in triplicate (*n* = 3). Statistical analysis was performed using one-way analysis of variance (ANOVA) followed by Tukey’s post hoc test. Different letters indicate statistically significant differences among groups within each condition (*p* < 0.05), whereas groups sharing at least one letter do not differ significantly.

Doxorubicin also induced apoptosis, increasing the percentages of early apoptotic cells (Annexin V+/PI–; 7.64 ± 0.66%) and late apoptotic/secondary necrotic cells (Annexin V+/PI+; 7.20 ± 0.67%). The combined percentage of these populations reached 14.84 ± 0.23% relative to the control group (*p* < 0.05 for all) (Figure 5).

### 2.4 CV2 Induces Oxidative Stress in MCF-7 Cells

The effect of CV2 on intracellular ROS production was evaluated using the DCFH-DA assay (Figure 6). After 30 min of treatment, a significant increase in the percentage of DCF-positive cells was observed only at 10 μM (21.20 ± 0.51%; *p* < 0.05) compared with the control group (1.85 ± 0.48%). After 3 h of exposure, both concentrations significantly increased ROS levels, reaching 37.55 ± 0.99% and 40.58 ± 2.47% DCF-positive cells at 5 and 10 μM, respectively, compared with 1.33 ± 0.18% in the corresponding control group (*p* < 0.05 for both).

**Figure 6:**
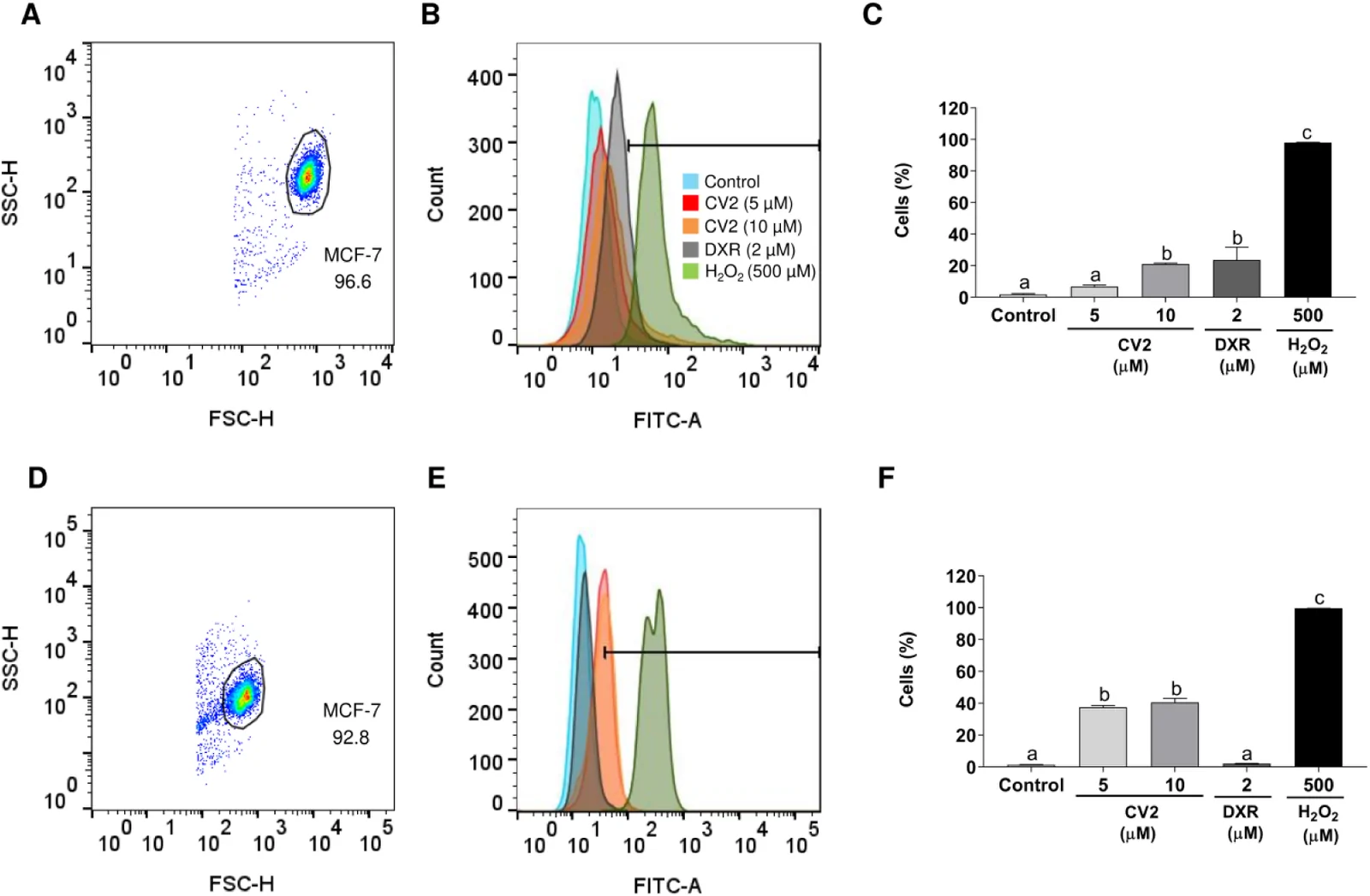
Effect of (E)-4-(1-epoxy-7,8-propen)­phenyl benzoate (CV2; 5 and 10 μM) and doxorubicin (DXR; 2 μM) on intracellular reactive oxygen species (ROS) levels in MCF-7 human breast adenocarcinoma cells after 30 min (A–C) and 3 h (D–F) of treatment, assessed using 2′,7′-dichlorofluorescein (DCF) fluorescence by flow cytometry. Representative dot plots (A and D), fluorescence histograms (B and E), and quantitative analysis of DCF fluorescence intensity (C and F) are shown. Data are presented as mean ± standard error of the mean (SEM) from three independent experiments performed in triplicate (*n* = 3). Statistical analysis was performed using one-way analysis of variance (ANOVA) followed by Tukey’s post hoc test. Different letters indicate statistically significant differences among groups (*p* < 0.05), whereas groups sharing at least one letter do not differ significantly.

Doxorubicin (2 μM) significantly increased ROS production after 30 min of treatment (23.70 ± 8.20%; *p* < 0.05), but not after 3 h. As expected, hydrogen peroxide (H_2_O_2_; 500 μM) markedly increased the percentage of DCF-positive cells at both time points, reaching 98.03 ± 0.21% after 30 min and 99.60 ± 0.08% after 3 h of exposure (*p* < 0.05) (Figure 6).

### 2.5 Oxidative Stress Modulates the Cytotoxicity of CV2 in MCF-7 Cells

Treatment with CV2 for 72 h significantly reduced the viability of MCF-7 cells in the absence of N-acetylcysteine (NAC; 5 mM). Cell viability decreased to 49.57 ± 1.91% and 27.23 ± 2.37% following treatment with 5 and 10 μM CV2, respectively, compared with 101.00 ± 5.77% in the control group (*p* < 0.05 for both) (Figure 7).

**Figure 7:**
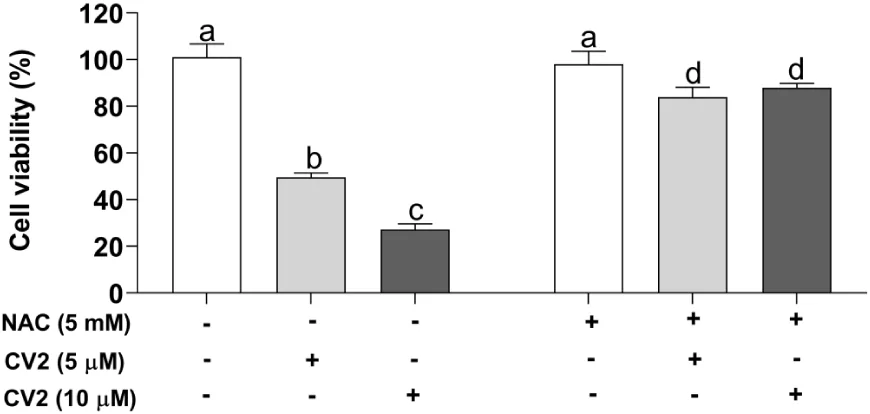
Effect of N-acetylcysteine (NAC; 5 mM) pretreatment on the cytotoxicity of (E)-4-(1-epoxy-7,8-propen)­phenyl benzoate (CV2; 5 and 10 μM) in MCF-7 human breast adenocarcinoma cells after 72 h of treatment, as determined by the MTT assay. Data are presented as mean ± standard error of the mean (SEM) from three independent experiments performed in triplicate (*n* = 3), and expressed as a percentage of cell viability (%). Statistical analysis was performed using one-way analysis of variance (ANOVA) followed by Tukey’s post hoc test. Different letters indicate statistically significant differences among groups (*p* < 0.05), whereas groups sharing at least one letter do not differ significantly.

Pretreatment for 3 h with NAC markedly attenuated the cytotoxic effect of CV2, increasing cell viability to 83.90 ± 4.29% and 87.90 ± 1.98% in cells treated with 5 and 10 μM CV2, respectively, compared with the corresponding groups not exposed to NAC (*p* < 0.05 for both). These results indicate that oxidative stress contributes to the cytotoxic activity induced by CV2 in MCF-7 cells (Figure 7).

### 2.6 The Cytotoxicity of CV2 Involves JNK and p38 MAPK Signaling

Pretreatment with the JNK inhibitor SP600125 significantly attenuated the cytotoxic effect of CV2 in MCF-7 cells. Cell viability increased from 47.06 ± 1.49% and 28.53 ± 1.61% in cells treated with 5 and 10 μM CV2 alone to 84.17 ± 2.54% and 82.80 ± 2.22%, respectively, in the presence of the inhibitor (*p* < 0.05 for both) (Figure 8A).

**Figure 8:**
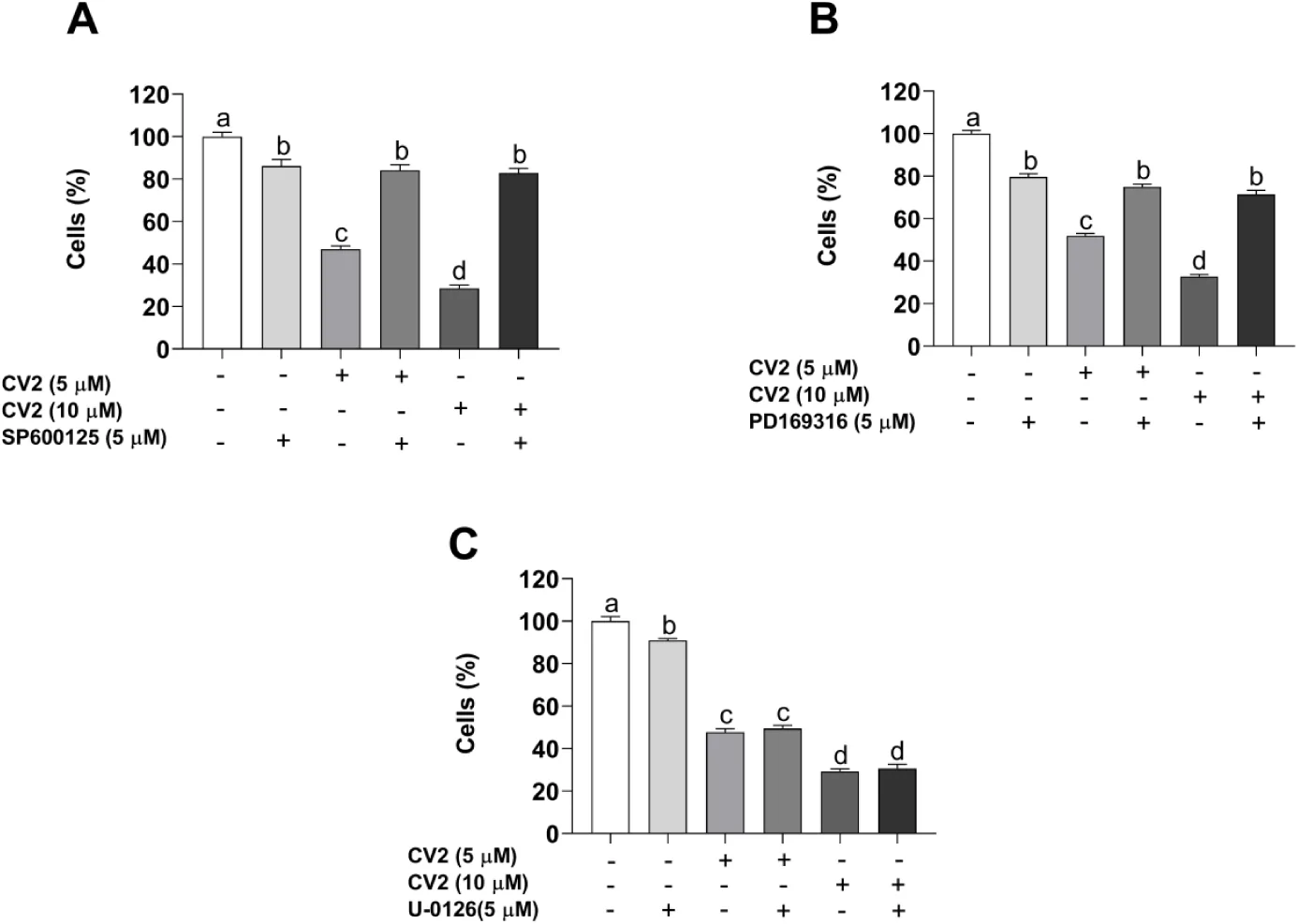
Effect of pharmacological inhibition of mitogen-activated protein kinase (MAPK) pathways on the cytotoxicity of (E)-4-(1-epoxy-7,8-propen)­phenyl benzoate (CV2) in MCF-7 human breast adenocarcinoma cells after 72 h of treatment, as determined by the MTT assay. Cells were pretreated for 3 h with (A) the c-Jun N-terminal kinase (JNK) inhibitor SP600125 (5 μM), (B) the p38 MAPK inhibitor PD169316 (5 μM), or (C) the extracellular signal-regulated kinase 1/2 (ERK1/2) inhibitor U-0126 (5 μM) prior to CV2 exposure (5 and 10 μM). Data are presented as mean ± standard error of the mean (SEM) from three independent experiments performed in triplicate (*n* = 3). Statistical analysis was performed using one-way analysis of variance (ANOVA) followed by Tukey’s post hoc test. Different letters indicate statistically significant differences among groups (*p* < 0.05), whereas groups sharing at least one letter do not differ significantly.

Similarly, pretreatment with the p38 MAPK inhibitor PD169316 significantly increased cell viability, from 51.91 ± 1.10% and 32.78 ± 0.92% in cells treated with 5 and 10 μM CV2 alone to 74.91 ± 1.37% and 71.50 ± 1.88%, respectively, in the presence of the inhibitor (*p* < 0.05 for both) (Figure 8B).

In contrast, pretreatment with the ERK1/2 inhibitor U0126 did not significantly affect the cytotoxic activity of CV2. Cell viability remained unchanged in comparison with the corresponding groups treated with CV2 alone (Figure 8C).

### 2.7 CV2 Modulates MAPK Signaling in MCF-7 Cells

Treatment with CV2 significantly increased the percentage of phospho-JNK1-positive cells, reaching 98.17 ± 0.46% and 98.00 ± 0.28% following exposure to 5 and 10 μM, respectively, compared with 0.14 ± 0.02% in the control group (*p* < 0.05 for both) (Figure 9).

**Figure 9:**
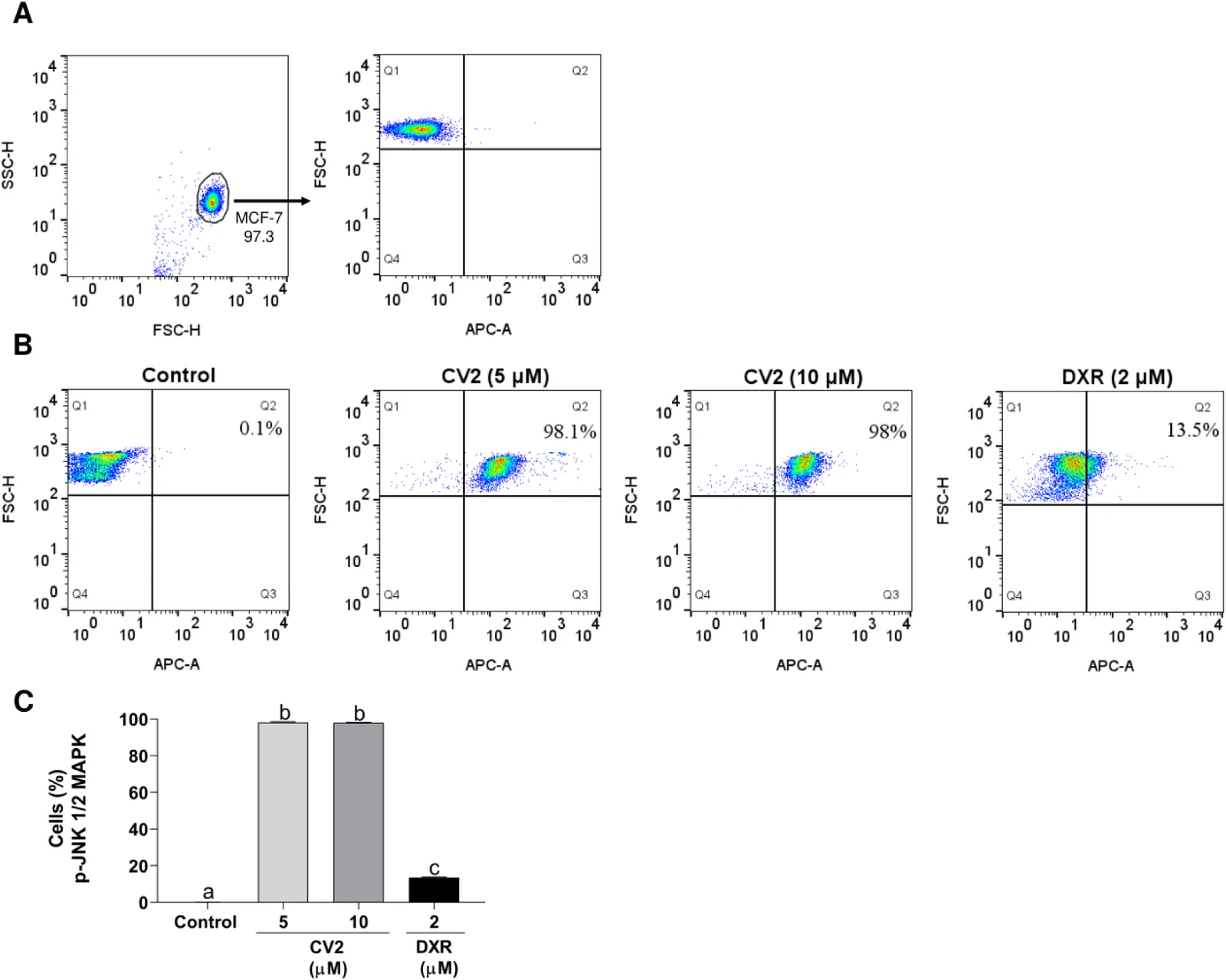
Effect of (E)-4-(1-epoxy-7,8-propen)­phenyl benzoate (CV2; 5 and 10 μM) and doxorubicin (DXR; 2 μM) on c-Jun N-terminal kinase 1 (JNK1) phosphorylation in MCF-7 human breast adenocarcinoma cells after 48 h of treatment, assessed by flow cytometry using an antiphospho-JNK1 antibody. Representative dot plots are shown together with the quantification of phospho-JNK1-positive cells. Data are presented as mean ± standard error of the mean (SEM) from three independent experiments performed in duplicate (*n* = 3). Statistical analysis was performed using one-way analysis of variance (ANOVA) followed by Tukey’s post hoc test. Different letters indicate statistically significant differences among groups (*p* < 0.05), whereas groups sharing at least one letter do not differ significantly.

Similarly, CV2 significantly increased the percentage of phospho-p38 MAPK-positive cells from 15.98 ± 0.76% in the control group to 93.88 ± 0.32% and 93.43 ± 1.42% after treatment with 5 and 10 μM, respectively (*p* < 0.05 for both) (Figure 10).

**Figure 10:**
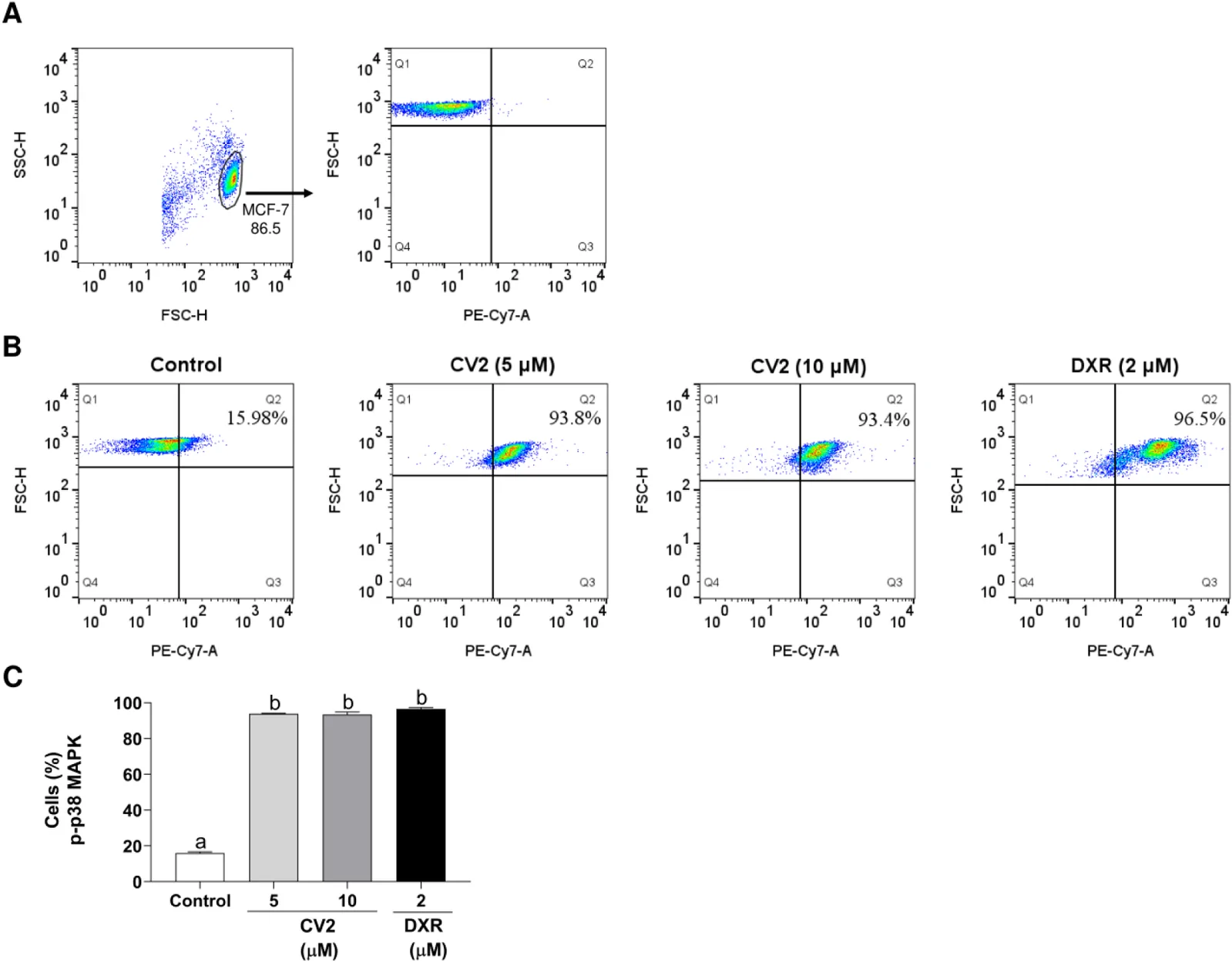
Effect of (E)-4-(1-epoxy-7,8-propen)­phenyl benzoate (CV2; 5 and 10 μM) and doxorubicin (DXR; 2 μM) on p38 Mitogen-Activated Protein Kinase (p38 MAPK) phosphorylation in MCF-7 human breast adenocarcinoma cells after 48 h of treatment, assessed by flow cytometry using an antiphospho-p38 antibody. Representative dot plots are shown together with the quantification of phospho-p38-positive cells. Data are presented as mean ± standard error of the mean (SEM) from three independent experiments performed in duplicate (*n* = 3). Statistical analysis was performed using one-way analysis of variance (ANOVA) followed by Tukey’s post hoc test. Different letters indicate statistically significant differences among groups (*p* < 0.05), whereas groups sharing at least one letter do not differ significantly.

CV2 also increased the percentage of phospho-ERK1/2-positive cells, from 1.18 ± 0.19% in the control group to 22.10 ± 1.22% and 34.10 ± 2.32% following treatment with 5 and 10 μM, respectively (*p* < 0.05 for both) (Figure 11).

**Figure 11:**
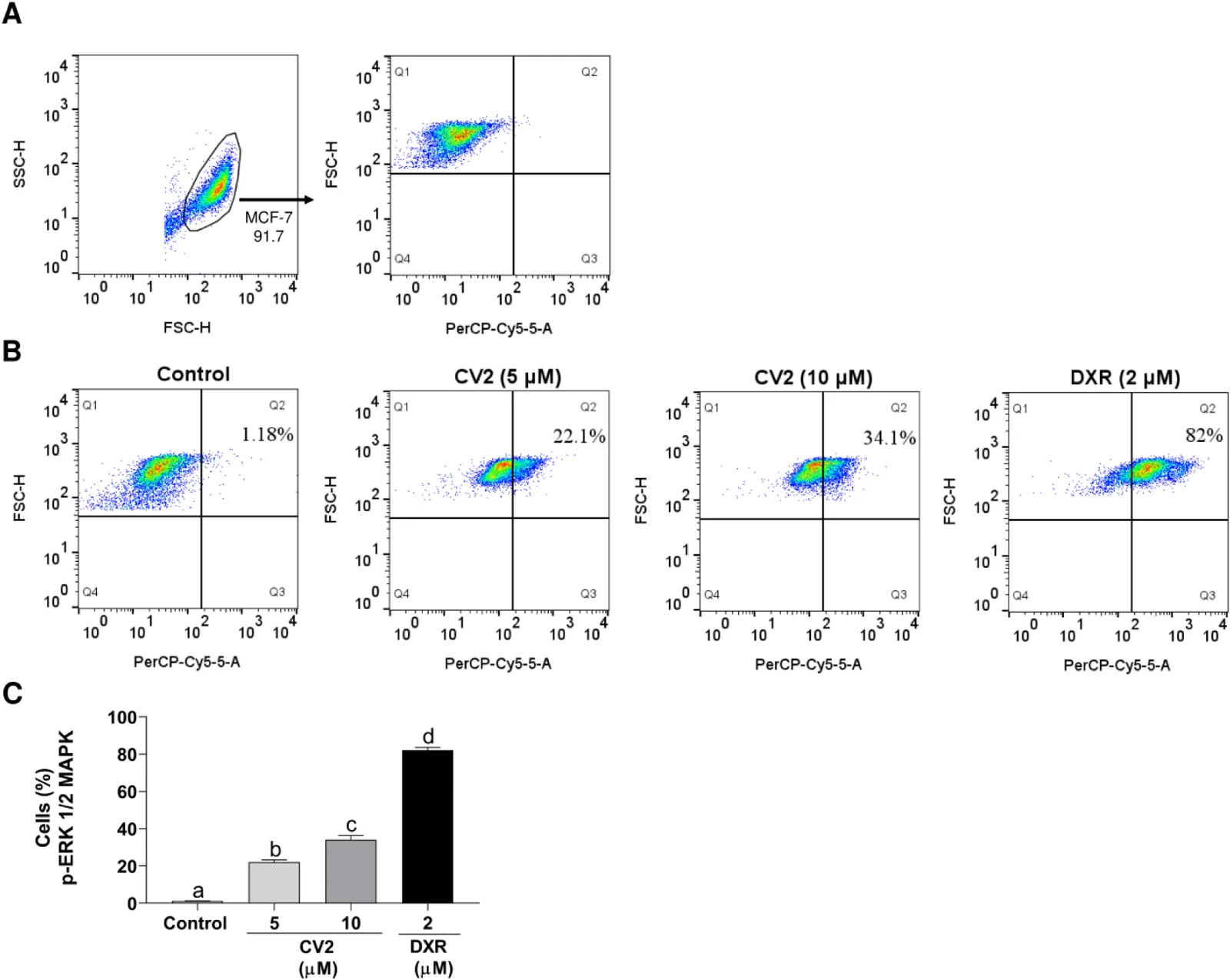
Effect of (E)-4-(1-epoxy-7,8-propen)­phenyl benzoate (CV2; 5 and 10 μM) and doxorubicin (DXR; 2 μM) on Extracellular Signal-Regulated Kinase 1 and 2 (ERK1/2) phosphorylation in MCF-7 human breast adenocarcinoma cells after 48 h of treatment, assessed by flow cytometry using an antiphospho-ERK1/2 antibody. Representative dot plots are shown together with the quantification of phospho-ERK1/2-positive cells. Data are presented as mean ± standard error of the mean (SEM) from three independent experiments performed in duplicate (*n* = 3). Statistical analysis was performed using one-way analysis of variance (ANOVA) followed by Tukey’s post hoc test. Different letters indicate statistically significant differences among groups (*p* < 0.05), whereas groups sharing at least one letter do not differ significantly.

Doxorubicin (2 μM) likewise increased the percentages of phospho-JNK1-positive (13.52 ± 0.46%), phospho-ERK1/2-positive (82.08 ± 1.62%), and phospho-p38 MAPK-positive cells (96.57 ± 0.83%) compared with their respective control groups (*p* < 0.05 for all) (Figure 9–11).

### 2.8 CV2 Increases PKB/AKT Phosphorylation in MCF-7 Cells

As shown in Figure 12, treatment with CV2 for 48 h significantly increased the percentage of phospho-PKB/AKT-positive cells, from 0.68 ± 0.16% in the control group to 83.03 ± 1.38% and 77.68 ± 0.65% following treatment with 5 and 10 μM, respectively (*p* < 0.05 for both). Doxorubicin (2 μM) also increased the percentage of phospho-PKB/AKT-positive cells, reaching 53.33 ± 1.80% (*p* < 0.05) compared with the control group (Figure 12).

**Figure 12:**
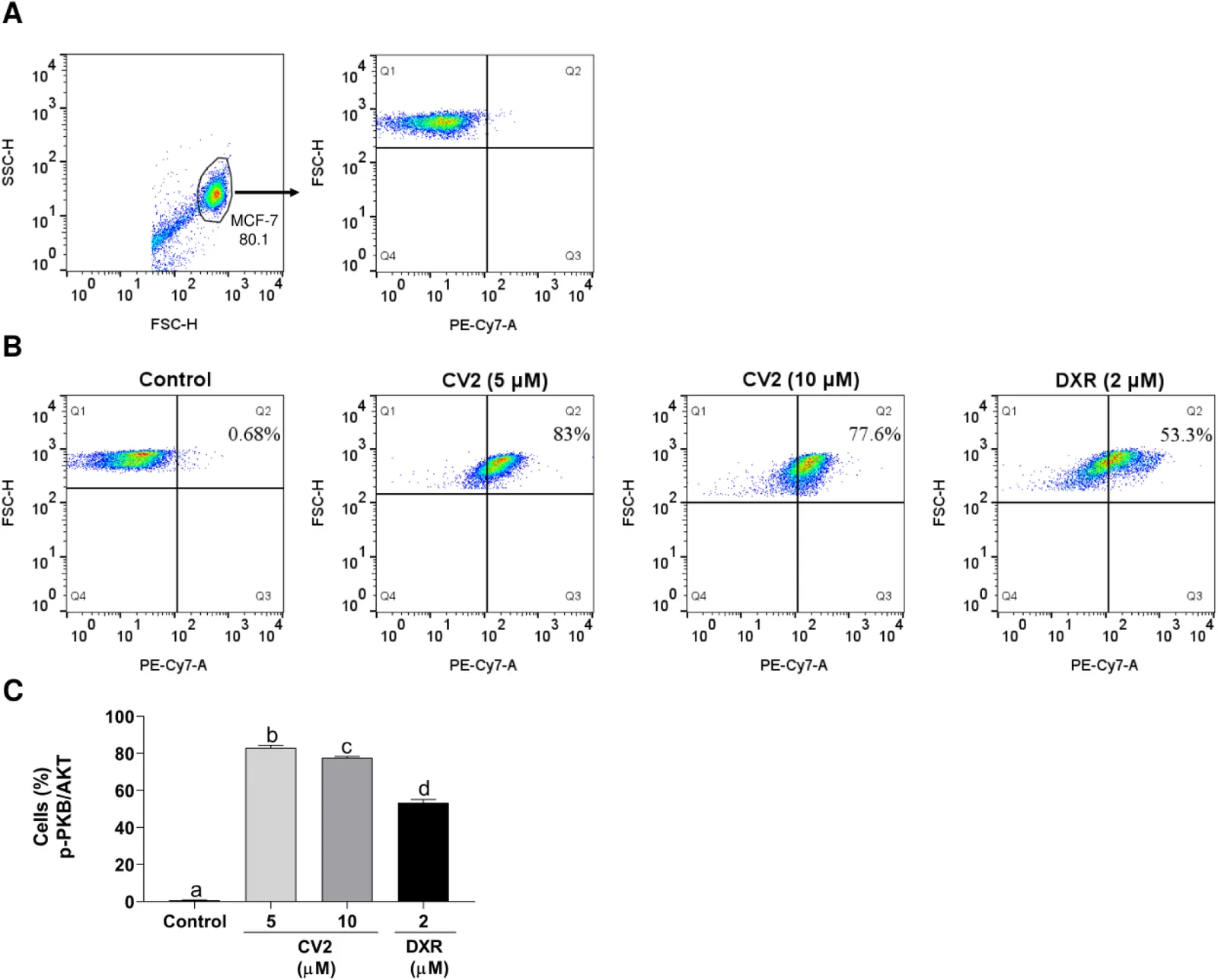
Effect of (E)-4-(1-epoxy-7,8-propen)­phenyl benzoate (CV2; 5 and 10 μM) and doxorubicin (DXR; 2 μM) on Protein Kinase B (PKB/AKT) phosphorylation in MCF-7 human breast adenocarcinoma cells after 48 h of treatment, assessed by flow cytometry using an antiphospho-PKB/AKT antibody. Representative dot plots are shown together with the quantification of phospho-PKB/AKT-positive cells. Data are presented as mean ± standard error of the mean (SEM) from three independent experiments performed in duplicate (*n* = 3). Statistical analysis was performed using one-way analysis of variance (ANOVA) followed by Tukey’s post hoc test. Different letters indicate statistically significant differences among groups (*p* < 0.05), whereas groups sharing at least one letter do not differ significantly.

### 2.9 CV2 Increases NF-κB Phosphorylation in MCF-7 Cells

Treatment with CV2 for 48 h significantly increased the percentage of phospho-NF-κB p65-positive cells, from 2.75 ± 0.36% in the control group to 17.87 ± 0.97% and 19.37 ± 0.81% following exposure to 5 and 10 μM, respectively (*p* < 0.05 for both). Doxorubicin (2 μM) also increased the percentage of phospho-NF-κB p65-positive cells, reaching 30.70 ± 1.04% (*p* < 0.05) compared with the control group (Figure 13).

**Figure 13:**
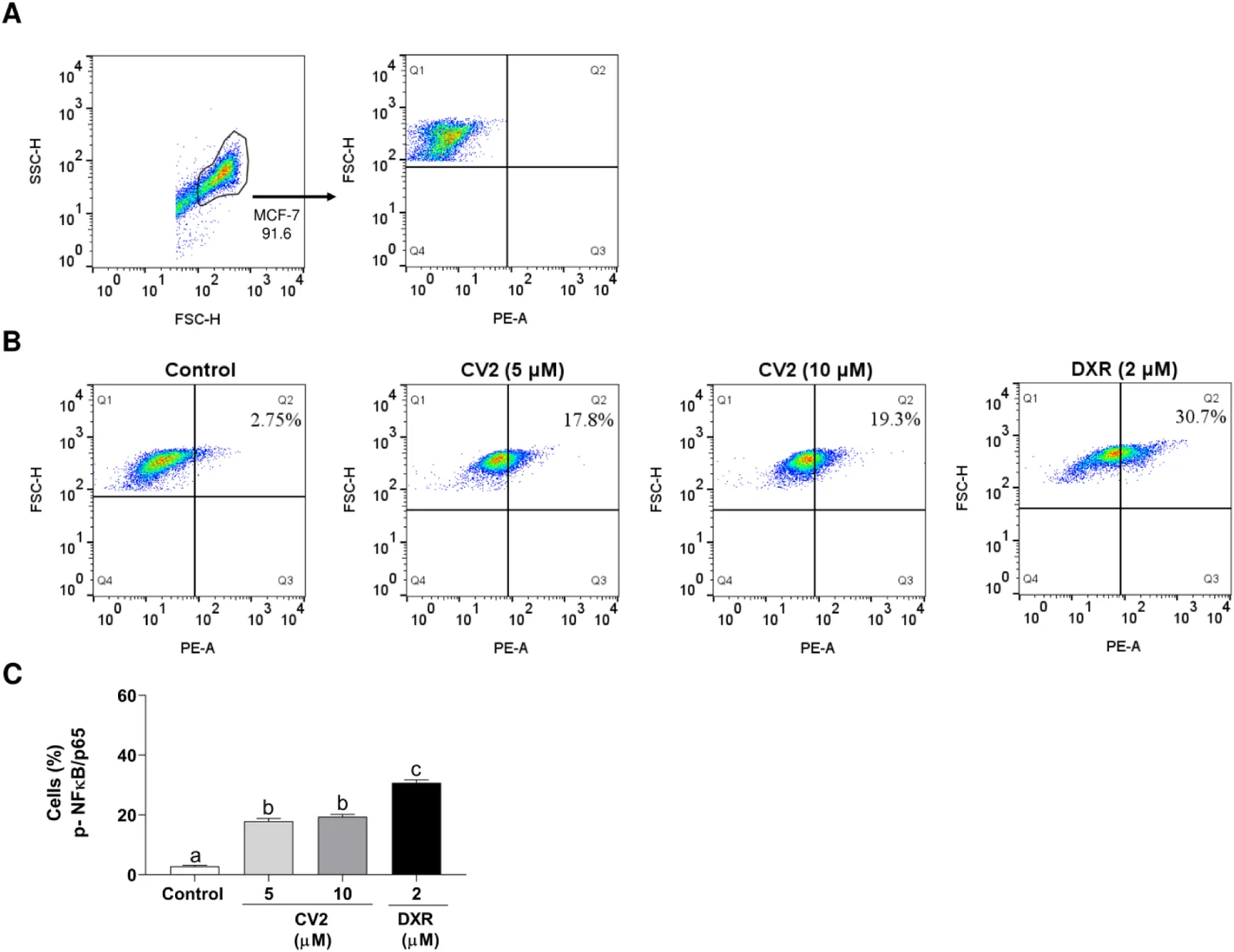
Effect of (E)-4-(1-epoxy-7,8-propen)­phenyl benzoate (CV2; 5 and 10 μM) and doxorubicin (DXR; 2 μM) on Nuclear Factor kappa B (NF-κB) p65 phosphorylation in MCF-7 human breast adenocarcinoma cells after 48 h of treatment, assessed by flow cytometry using an antiphospho-NF-κB p65 antibody. Representative dot plots are shown together with the quantification of phospho-NF-κB p65-positive cells. Data are presented as mean ± standard error of the mean (SEM) from three independent experiments performed in duplicate (*n* = 3). Statistical analysis was performed using one-way analysis of variance (ANOVA) followed by Tukey’s post hoc test. Different letters indicate statistically significant differences among groups (*p* < 0.05), whereas groups sharing at least one letter do not differ significantly.

### 2.10 Embryotoxicity of CV2 in the Zebrafish Model

Exposure of zebrafish embryos to CV2 for 96 h resulted in concentration-dependent embryotoxicity (Figure 14). The highest concentration tested (4 μM) caused 100% mortality within the first 24 h of exposure, whereas lethality decreased progressively at lower concentrations. No mortality or nonlethal effects were observed in embryos exposed to 2 μM CV2. The median lethal concentration (LC_50_) of CV2 was estimated at 3.07 μM (95% CI: 2.886–3.275 μM). Morphological evaluation revealed egg coagulation in embryos exposed to 4 μM CV2 after 24 h of treatment, whereas no visible abnormalities were detected at lower concentrations (Figure 15).

**Figure 14:**
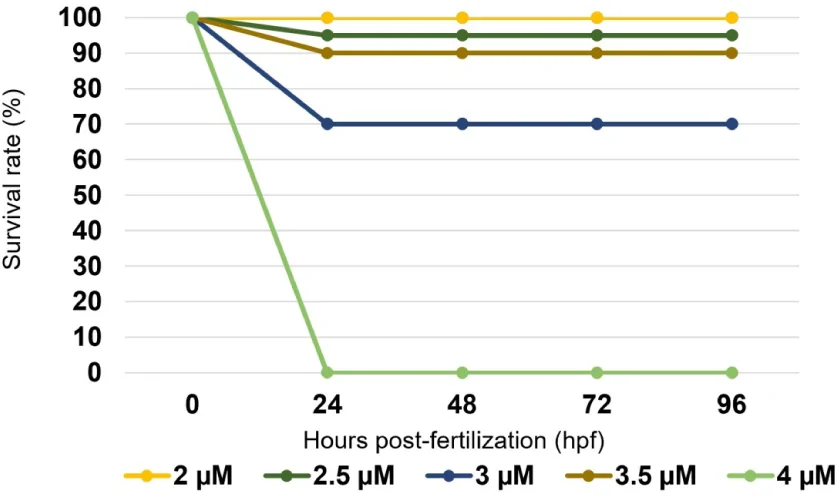
Survival of zebrafish (*Danio rerio*) embryos exposed to increasing concentrations of (E)-4-(1-epoxy-7,8-propen)­phenyl benzoate (CV2) during the Fish Embryo Toxicity (FET) assay. Embryos up to 3 h postfertilization (hpf) were exposed to CV2 for 96 h under static conditions, and survival was monitored every 24 h according to Organization for Economic Co-operation and Development (OECD) Guideline No. 236. Each concentration was evaluated using 20 embryos, and survival rates are expressed as the percentage of viable embryos at each observation time.

**Figure 15:**
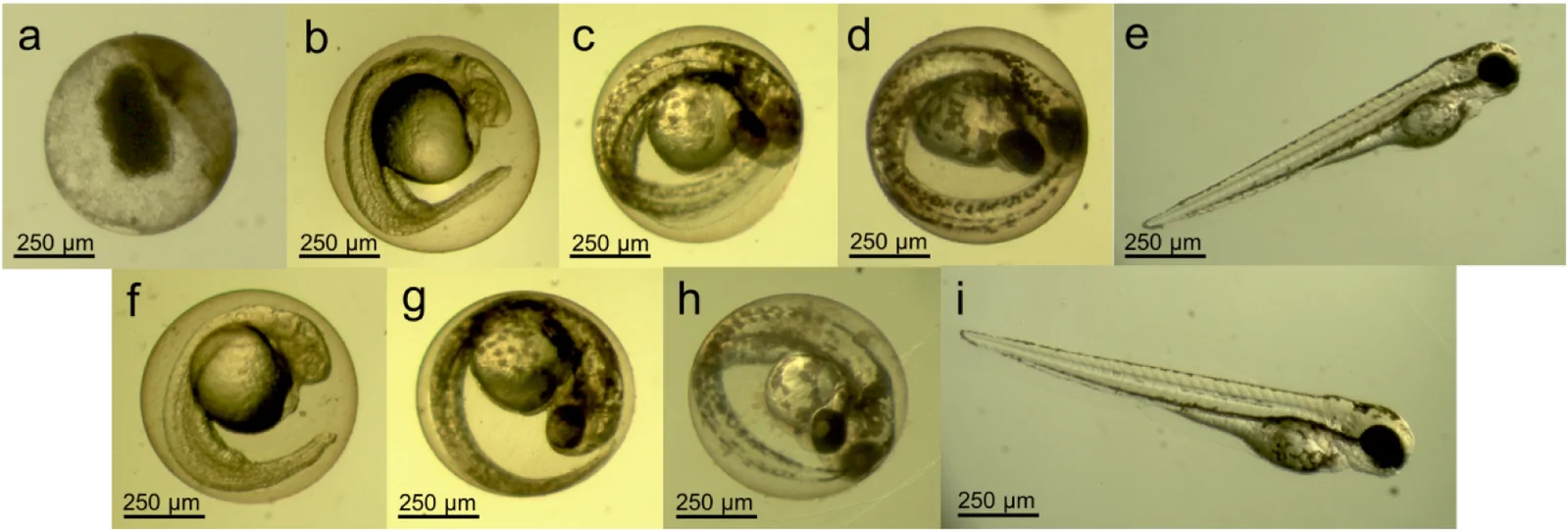
Representative images of lethal and nonlethal effects observed in zebrafish (*Danio rerio*) embryos during the Fish Embryo Toxicity (FET) assay following exposure to (E)-4-(1-epoxy-7,8-propen)­phenyl benzoate (CV2). (a) Embryo exposed to 4 μM CV2 showing egg coagulation at 24 h postfertilization (hpf), a lethal end point according to Organisation for Economic Co-operation and Development (OECD) Guideline No. 236. (b–e) Embryos exposed to 2.5 μM CV2 exhibiting normal development at 24, 48, 72, and 96 hpf, respectively. (f–i) Negative control embryos maintained in E3 medium, showing normal development at 24, 48, 72, and 96 hpf, respectively.

### 2.11 Effects of CV2 on Oxidative Stress-Related Enzymes in Zebrafish Larvae

Additionally, a significant reduction in (CAT) activity was observed in embryos treated with all three concentrations (0.5 μM: 2.93 ± 0.25 μmol/min/mg; 1.0 μM: 2.26 ± 0.28 μmol/min/mg; and 2.0 μM: 2.54 ± 0.31 μmol/min/mg, *p* < 0.05 for all), compared to the control group (3.44 ± 0.24 μmol/min/mg). (Figure 16).

**Figure 16:**
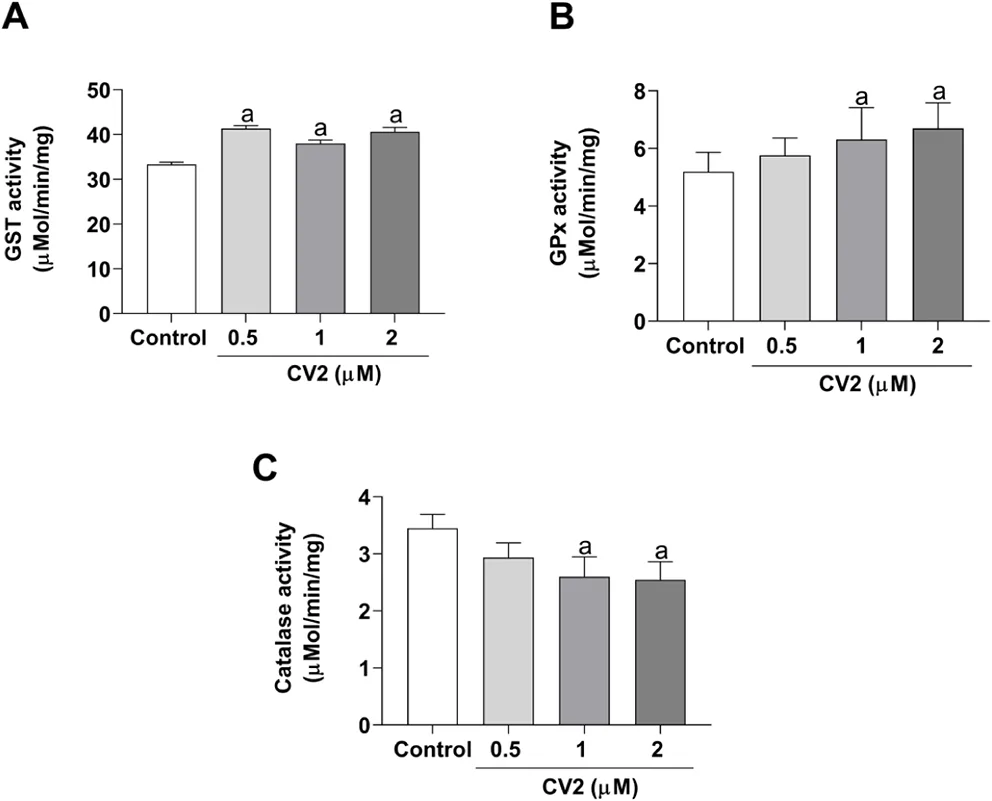
Effect of (E)-4-(1-epoxy-7,8-propen)­phenyl benzoate (CV2) on antioxidant enzyme activities in zebrafish (*Danio rerio*) larvae after 96 h of exposure. Activities of (A) glutathione S-transferase (GST), (B) glutathione peroxidase (GPx), and (C) catalase (CAT) were determined in larvae exposed to sublethal concentrations of CV2 (0.5, 1.0, and 2.0 μM). Data are presented as mean ± standard error of the mean (SEM), and enzyme assays were performed in quadruplicate for each experimental group. Statistical analysis was performed using one-way analysis of variance (ANOVA) followed by Tukey’s post hoc test. *p* < 0.05 compared with the control group.

After 96 h of exposure, CV2 significantly altered the activity of antioxidant enzymes in zebrafish larvae (Figure 16). Glutathione S-transferase (GST) activity increased at all tested concentrations (0.5, 1.0, and 2.0 μM), reaching 41.34 ± 0.68, 38.02 ± 0.74, and 40.64 ± 0.95 μmol/min/mg, respectively, compared with 33.36 ± 0.48 μmol/min/mg in the control group (*p* < 0.05 for all).

Similarly, glutathione peroxidase (GPx) activity was significantly increased following exposure to CV2, with values of 5.76 ± 0.60, 6.31 ± 0.90, and 6.69 ± 0.88 μmol/min/mg at 0.5, 1.0, and 2.0 μM, respectively, compared with 5.18 ± 0.67 μmol/min/mg in the control group (*p* < 0.05 for all).

In contrast, catalase (CAT) activity was significantly reduced in larvae exposed to CV2, decreasing from 3.44 ± 0.24 μmol/min/mg in the control group to 2.93 ± 0.25, 2.26 ± 0.28, and 2.54 ± 0.31 μmol/min/mg at 0.5, 1.0, and 2.0 μM, respectively (*p* < 0.05 for all) (Figure 16).

## 3 Discussion

Breast cancer remains a major global health challenge, highlighting the need for the development of more effective and selective therapeutic agents.^33,34^ In this context, natural products and their derived phytochemicals have emerged as valuable sources of bioactive compounds with anticancer potential.^35–37^ Among them, the phenylpropanoid (E)-4-(1-epoxy-7,8-propen)­phenyl benzoate (CV2), isolated from the roots of *Croton velutinus* (Euphorbiaceae), was previously shown to exert cytotoxic activity against MCF-7 breast cancer cells.^8^ Building upon these findings, the present study further characterized the antitumor effects of CV2 by evaluating its cytotoxicity in human tumor and nontumor cell lines, as well as its effects on cell cycle progression, apoptosis, oxidative stress, and signaling pathways associated with MAPKs, NF-κB, and PKB/AKT in MCF-7 cells.

The antitumor activity of CV2 was evaluated in a panel of human tumor cell lines, including HCT-116 (colon carcinoma), HeLa (cervical carcinoma), SK-MEL-28 (melanoma), MDA-MB-231 (triple-negative breast carcinoma), and MCF-7 (luminal breast carcinoma), using doxorubicin (DXR) as the reference drug. CV2 exhibited cytotoxic activity against all tumor cell lines tested, with MCF-7 cells showing the highest sensitivity to treatment, corroborating previous findings reported by Abreu et al. (2020). The greater sensitivity of MCF-7 cells may reflect cell-specific differences in redox homeostasis, apoptotic signaling, drug metabolism, or the regulation of pro-survival pathways, highlighting the influence of cellular context on the response to CV2. These findings reinforce the relevance of phenylpropanoids in the search for novel breast cancer therapeutics, as multiple members of this class have been reported to exert significant cytotoxic effects in breast cancer models.^38–41^

Selectivity toward tumor cells is a desirable characteristic for anticancer drug candidates. Importantly, CV2 displayed a selective cytotoxic effect toward breast cancer cells, exhibiting substantially lower toxicity in the nontumorigenic MCF-10A cell line. This selectivity profile, reflected by a high selectivity index, highlights the potential of CV2 as a candidate for further anticancer investigation.

To further investigate its toxicity profile, the effects of CV2 were evaluated in human peripheral blood mononuclear cells (PBMCs), which are frequently used as a nontumor model in the preclinical assessment of anticancer compounds.^42–44^ Although CV2 reduced PBMC viability, its cytotoxic effect was markedly lower than that of doxorubicin, suggesting a more favorable *in vitro* safety profile than the reference drug. Similar evaluations performed with other phenylpropanoid derivatives have demonstrated relatively low toxicity toward PBMCs.^45,46^ It is noteworthy that the greater toxicity of DXR toward PBMCs is consistent with the well-established hematological toxicity associated with conventional chemotherapeutic agents, including myelosuppression.^47,48^ Together, these findings indicate that CV2 combines potent cytotoxic activity against breast cancer cells with a favorable selectivity profile in nontumor cellular models. Additional information regarding the toxicity profile of CV2 was obtained using the zebrafish model and will be discussed in a subsequent section.

Having established the cytotoxic activity and selectivity of CV2 toward breast cancer cells, subsequent experiments were designed to investigate the mechanisms underlying its antitumor effects. To this end, we evaluated whether CV2-induced cytotoxicity was associated with alterations in cell cycle progression, apoptosis induction, oxidative stress, and the modulation of signaling pathways known to regulate cell fate decisions, including MAPKs, NF-κB, and PKB/AKT.

Cell cycle deregulation is a hallmark of cancer and plays a central role in sustaining the uncontrolled proliferation of malignant cells.^49,50^ In the present study, treatment with CV2 significantly increased the sub-G1 population, a well-established indicator of apoptotic DNA fragmentation.^51–53^ Similar findings have been reported for other phenylpropanoids in different cancer models. For example, p-coumaric acid and verbascoside increased the sub-G1 population in HCT-15 and MKN45 cells, respectively.^54,55^

Because accumulation of cells in the sub-G1 region is commonly associated with apoptotic DNA fragmentation, we next evaluated whether CV2-induced cytotoxicity involved apoptotic cell death. Resistance to apoptosis is a hallmark of cancer and represents a major obstacle to successful anticancer therapy, contributing to tumor progression, metastasis, and treatment resistance.^50,56^ In the present study, the increase in the sub-G1 population was accompanied by a marked increase in early apoptotic cells (Annexin V+/PI−), supporting the involvement of apoptosis in the cytotoxic activity of CV2 in MCF-7 cells. CV2 also increased the proportion of Annexin V+/PI+ cells, which may represent cells in late apoptosis and/or secondary necrosis, as these populations cannot be unequivocally distinguished by Annexin V/PI staining alone. Thus, the concomitant detection of DNA fragmentation and phosphatidylserine externalization provides complementary evidence supporting the involvement of apoptotic cell death in the cytotoxic effects of CV2, while the increased Annexin V+/PI+ population may reflect progression toward late apoptosis and/or secondary necrosis.

The ability to trigger apoptosis has been widely reported for phenylpropanoids and is considered one of the main mechanisms responsible for their antitumor activity. For example, resveratrol induces apoptosis in 4T1 murine mammary carcinoma cells, whereas eugenol promotes apoptotic cell death in human breast cancer (MCF-7 and MDA-MB-231) and melanoma (A2058) cell lines.^57–59^ Together, these findings indicate that the potent cytotoxic activity of CV2 against MCF-7 cells is closely associated with the activation of apoptotic pathways, further supporting the potential of this phenylpropanoid as a promising anticancer agent.

Reactive oxygen species (ROS) are important regulators of cancer cell biology. While moderate elevations in ROS can support tumor progression through the induction of genomic instability, DNA damage, and activation of pathways involved in cell proliferation, survival, and therapeutic resistance, excessive ROS accumulation may exceed the antioxidant capacity of cancer cells, ultimately triggering oxidative damage and cell death pathways.^60,61^

The data obtained herein showed that CV2 significantly increased intracellular ROS levels in MCF-7 cells, as demonstrated by the DCFH-DA assay. A key finding of this study was that pretreatment with the antioxidant N-acetylcysteine (NAC) markedly attenuated the cytotoxic effect of CV2, providing functional evidence that oxidative stress plays a central role in its antitumor activity.

The involvement of ROS in the cytotoxic effects of phenylpropanoids has been reported in several cancer models. For example, syringin, a phenylpropanoid isolated from *Eleutherococcus senticosus*, increased ROS levels in MCF-7 and MDA-MB-231 cells, whereas NAC significantly reduced its cytotoxic activity.^62^ Similarly, asaricin and isoasarone, isolated from *Piper sarmentosum*, induced oxidative stress associated with cell cycle disruption and apoptosis in MDA-MB-231 cells.^63^ Eugenol has also been shown to increase ROS production and promote apoptosis in breast cancer, cervical cancer, and melanoma cell lines.^59^

Taken together, these findings indicate that ROS generation is not merely a consequence of CV2 exposure but rather an important event contributing to its cytotoxic activity. The ability of NAC to significantly reverse CV2-induced cytotoxicity further supports the involvement of oxidative stress as a key mechanism underlying the antitumor effects of this phenylpropanoid.

ROS are well-established modulators of intracellular signaling pathways, including MAPKs, PKB/AKT, and NF-κB.^64,65^ Therefore, considering the marked increase in intracellular ROS levels induced by CV2 and the ability of oxidative stress to regulate signaling networks involved in cell survival and death, we investigated whether the cytotoxic effects of this compound were associated with alterations in these pathways.

MAPKs comprise a family of serine/threonine kinases that includes ERK1/2, JNK, and p38 MAPK, which regulate diverse cellular processes such as proliferation, differentiation, survival, stress responses, and apoptosis.^12^ In cancer cells, the biological consequences of MAPK activation are highly context-dependent and may result in either pro-survival or pro-apoptotic responses. These outcomes are influenced by several factors, including the nature of the stimulus, the intensity and duration of signaling, the MAPK isoforms involved, the activity of upstream and downstream regulatory proteins, phosphatase-mediated negative feedback mechanisms, and post-translational modifications that modulate pathway activity.^16,66–70^ Our findings demonstrated that CV2 increased the phosphorylation of ERK1/2, JNK, and p38 MAPK. However, pharmacological inhibition experiments revealed distinct functional roles for these proteins. While inhibition of JNK and p38 significantly attenuated CV2-induced cytotoxicity, ERK inhibition had no significant effect on cell viability. These findings suggest that JNK and p38, but not ERK1/2, contribute to the cytotoxic activity of CV2 in MCF-7 cells.

The involvement of MAPK signaling in the antitumor activity of phenylpropanoids has been documented.^71^ Methyl ferulate increased JNK phosphorylation in A549 lung carcinoma cells,^39^ whereas capsaicin-induced apoptosis in pancreatic cancer cells was associated with ROS generation and JNK activation.^72^ Similarly, cinnamic acid increased the expression of JNK, p38 MAPK, and ERK in HepG2 cells, while salidroside promoted JNK-dependent antitumor effects in HT29 colorectal cancer cells.^73–76^ Collectively, these studies support a role for MAPK modulation in the anticancer activity of this class of compounds. Consistent with these observations, our results suggest that oxidative stress induced by CV2 is associated with activation of MAPK signaling, particularly JNK and p38, which appear to contribute to the apoptotic and cytotoxic effects observed in MCF-7 cells. Notably, the present findings are further supported by previous molecular docking analyses performed by our group, which demonstrated favorable interactions between CV2 and ERK, JNK, and p38 proteins.^9^ Although docking studies do not establish causality, the convergence of computational predictions, phospho-protein analyses, and pharmacological inhibition assays strengthens the evidence that MAPK signaling contributes to the antitumor activity of CV2.

PKB/AKT signaling is primarily recognized as a pro-survival pathway involved in the regulation of cell growth, proliferation, metabolism, and therapeutic resistance.^77–79^ Consequently, inhibition of AKT has been extensively explored as a strategy to enhance the efficacy of anticancer therapies, and numerous natural products have been screened for their ability to suppress this pathway.^80,81^ Consistent with its well-established role in tumor cell survival, several conventional chemotherapeutic agents, including doxorubicin, cisplatin, and paclitaxel, have been shown to induce AKT activation following treatment. Rather than contributing directly to cell death, this response is generally interpreted as a compensatory survival mechanism triggered by cellular stress, including oxidative damage, DNA damage, and other cytotoxic insults.^82–88^ This adaptive activation of AKT is considered one of the mechanisms by which cancer cells attempt to counteract the effects of anticancer therapies, providing the rationale for combining cytotoxic agents with AKT inhibitors in order to enhance therapeutic efficacy.^81,89–91^

However, the biological consequences of AKT activation appear to be highly context-dependent. Increasing evidence indicates that, under specific conditions, particularly those involving sustained oxidative stress, AKT activation may coexist with or contribute to apoptotic responses.^19,39,92,93^ In line with these observations, CV2 increased AKT phosphorylation while simultaneously inducing oxidative stress and apoptosis.

A phenomenon similar to that observed for PKB/AKT was detected in the present study also for NF-κB, as CV2 significantly increased NF-κB p65 phosphorylation despite inducing oxidative stress and apoptosis in MCF-7 cells. NF-κB is a transcription factor that regulates the expression of genes involved in inflammation, cell survival, proliferation, angiogenesis, invasion, metastasis, and therapeutic resistance.^94,95^ NF-κB activation is frequently associated with the expression of antiapoptotic genes and the establishment of a pro-tumorigenic microenvironment, making this pathway an attractive target for anticancer drug development. Nevertheless, the biological consequences of NF-κB activation are also highly context-dependent and may vary according to the cellular environment, the nature of the activating stimulus, and its crosstalk with other signaling pathways.^96–99^ In this context, several studies have reported NF-κB activation in association with apoptosis induced by cytotoxic compounds.^10,21,31,100,101^

Conversely, NF-κB activation may also represent an adaptive response to CV2-induced cellular stress, as this transcription factor is known to be activated by ROS and other cytotoxic stimuli. Given its established role in promoting survival pathways and limiting treatment-induced cell death, NF-κB has emerged as an attractive therapeutic target, and the combination of conventional anticancer agents with NF-κB inhibitors has been proposed as a promising strategy to improve antitumor efficacy.^102–104^

Taken together, the findings obtained for PKB/AKT and NF-κB indicate that CV2 modulates signaling pathways commonly associated with cell survival and stress adaptation. Although the precise functional significance of AKT and NF-κB activation remains to be fully elucidated, the concomitant induction of oxidative stress, apoptosis, and phosphorylation of both proteins suggests that these pathways may participate in the complex cellular response triggered by CV2. Whether their activation contributes to the cytotoxic effects of this phenylpropanoid, represents a compensatory mechanism aimed at limiting cellular damage, or reflects a combination of both processes remains unclear. Therefore, further mechanistic studies will be required to determine whether these pathways promote, oppose, or simply accompany the cytotoxic response induced by CV2.

The zebrafish Fish Embryo Toxicity (FET) assay is widely used as an early vertebrate model for the evaluation of compound toxicity and developmental effects.^105–107^ In the present study, exposure to CV2 resulted in concentration-dependent toxicity, which indicate that this phenylpropanoid exerts biological activity not only in tumor cells but also in a complex multicellular organism.

Comparable toxic effects have been reported for other natural products and phenylpropanoid-rich preparations. Shiau et al. (2021) demonstrated that exposure to curcumin at concentrations of 7.5 and 12.5 μM resulted in complete mortality of zebrafish embryos and larvae after 3 days of incubation.^108^ Likewise, an aqueous extract of *Piper sarmentosum*, rich in phenolic compounds including phenylpropanoids, significantly reduced embryo survival and impaired cardiac function during development.^109^ Eugenol has also been reported to induce embryotoxicity, causing delayed hatching and increased mortality in zebrafish embryos.^110^ Together, these findings indicate that developmental toxicity is not uncommon among bioactive phenolic compounds exhibiting relevant pharmacological activities.

When the toxicity-related end points evaluated in the present study are considered collectively, including PBMCs, MCF-10A cells, and zebrafish embryos, the safety profile of CV2 should be interpreted cautiously. Although the compound displayed potent cytotoxic activity against MCF-7 breast cancer cells, the proximity between the zebrafish LC_50_ and the concentrations required to induce antitumor effects suggests that its therapeutic window remains to be further defined. Nevertheless, it should be emphasized that the FET assay represents an early whole-organism screening model and does not directly predict therapeutic exposure limits in mammals. Similarly, PBMCs are highly responsive primary human cells that may exhibit increased sensitivity to cytotoxic and oxidative insults under *in vitro* conditions. The inclusion of MCF-10A cells, a nontumorigenic mammary epithelial model, provided additional evidence of selectivity within a tissue-relevant context. Therefore, while the present findings support the significant biological activity of CV2, further toxicological investigations using mammalian models will be necessary to establish its therapeutic index, safety profile, and translational potential.

The modulation of antioxidant enzymes observed in zebrafish larvae further supports the involvement of oxidative stress in the biological effects induced by CV2. Exposure to the compound resulted in reduced catalase (CAT) activity, a finding that may reflect increased oxidative burden, as CAT reduction is commonly associated with elevated intracellular ROS levels.^111,112^

In contrast, glutathione peroxidase (GPx) and glutathione S-transferase (GST) activities were significantly increased, suggesting the activation of compensatory antioxidant defense mechanisms aimed at limiting ROS-mediated damage. This pattern is consistent with the physiological role of GPx and GST in detoxification and maintenance of redox homeostasis under oxidative stress conditions. Similar responses have been reported for other natural products.^113–115^

Collectively, these findings are in agreement with the increase in intracellular ROS levels observed in MCF-7 cells and support oxidative stress as a central component of the biological activity of CV2. Moreover, the concomitant increase in GPx and GST activities suggests that zebrafish larvae were able to mount an adaptive antioxidant response to counterbalance the oxidative challenge imposed by the compound, at least under the sublethal exposure conditions evaluated in this study.

## 4 Materials and Methods

### 4.1 Drugs and Reagents

The following reagents were purchased from Sigma-Aldrich (St. Louis, MO, USA): Dulbecco’s Modified Eagle’s Medium (DMEM), Roswell Park Memorial Institute 1640 medium (RPMI-1640), Histopaque-1077, phosphate-buffered saline (PBS), doxorubicin (DXR), penicillin–streptomycin, 2′,7′-dichlorodihydrofluorescein diacetate (DCFH-DA), N-acetylcysteine (NAC), hydrogen peroxide (H_2_O_2_), 3-(4,5-dimethylthiazol-2-yl)-2,5-diphenyltetrazolium bromide (MTT), sodium chloride (NaCl), potassium chloride (KCl), calcium chloride (CaCl_2_), magnesium sulfate (MgSO_4_), sodium sulfate (Na_2_SO_4_), U0126, SP600125, PD169316, Triton X-100, Annexin V–FITC, and propidium iodide (PI). Dimethyl sulfoxide (DMSO) and sodium dodecyl sulfate (SDS) were purchased from Dinâmica (Indaiatuba, SP, Brazil) and Êxodo Científica (Sumaré, SP, Brazil), respectively. Phytohemagglutinin, trypsin (0.25%) containing ethylenediaminetetraacetic acid (EDTA), and fetal bovine serum (FBS) were obtained from Gibco (Grand Island, NY, USA). The following antibodies were obtained from BD Biosciences (San Jose, CA, USA): BD Phosflow Alexa Fluor 488 mouse anti-p-AKT (pS473), BD Phosflow PE mouse anti-p-NF-κB p65 (pS529), BD Phosflow PerCP-Cy 5.5 mouse anti-p-ERK1/2 (pT202/pY204), BD Phosflow Alexa Fluor 647 mouse anti-p-JNK (pT183/pY185), and BD Phosflow PE-Cy7 mouse anti-p-p38 MAPK (pT180/pY182).

The drugs and reagent solutions were prepared immediately before use.

### 4.2 Cell Lines

The tumor cell lines MCF-7 (human breast cancer), SK-MEL-28 (human melanoma), HeLa (human cervical cancer), HCT-116 (human colon carcinoma), MDA-MB-231 (triple-negative human breast cancer), and nontumorigenic human breast epithelial cell line MCF-10A were obtained from the Rio de Janeiro Cell Bank (BCRJ), RJ, Brazil, and cultured in Dulbecco’s Modified Eagle’s Medium (DMEM) (MCF-7, MDA-MB-231, SK-MEL-28, HeLa, MCF-10A) or Roswell Park Memorial Institute 1640 (RPMI) (HCT-116) medium supplemented with 10% fetal bovine serum and 1% penicillin-streptomycin, at 37 °C with 5% CO_2_.

### 4.3 Human Peripheral Blood Mononuclear Cells (PBMCs)

The study involving PBMCs was conducted in accordance with the Declaration of Helsinki and approved by the Ethics and Research Committee of University Hospital Lauro Wanderley, UFPB (protocol code 3.715.689). Peripheral blood samples (20 – 25 mL) were collected by venipuncture into EDTA-containing tubes from three healthy female donors, aged 20–32 years, and each donor was considered an independent biological experiment.

Human peripheral blood mononuclear cells (PBMCs) were isolated by density-gradient centrifugation using Histopaque-1077 (400 × g, 20 °C, for 30 min). The mononuclear cell layer was collected, washed with phosphate-buffered saline (PBS) (400 × g, 20 °C, 10 min), and resuspended in RPMI-1640 medium supplemented with 10% fetal bovine serum (FBS), 1% antibiotic solution (10,000 units/mL penicillin and 10 mg/mL streptomycin) and 2% phytohemagglutinin. Cells were seeded in 96-well plates at a density of 1 × 10^6^ cells/mL (100 μL per well) and incubated for 24 h at 37 °C in a humidified atmosphere containing 5% CO_2_.^43,116^ Subsequently, PBMCs were exposed to CV2 (1.25–20 μM) or doxorubicin (DXR; 0.15–5 μM) for 72 h. For each donor, concentration–response curves were generated independently using triplicate measurements at each concentration. IC_50_ values were determined by nonlinear regression, and the final results are expressed as mean ± standard error of the mean (SEM) across the three donors.

### 4.4 Botanical Material and Extraction/Isolation of the Phenylpropanoid (E)-4-(1-Epoxy-7,8-propen)­phenyl Benzoate (CV2)

Specimens of *Croton velutinus* (Euphorbiaceae) were harvested in the city of Morro do Chapéu (BA, Brazil) (11°33′00″S, 41°09′22″W) and registered in the National Management System of Genetic Patrimony and Associated Traditional Knowledge (SisGen) with access number A22E9B0. The species was identified by Dr. Maria Lenise Guedes, with a specimen deposited in the Alexandre Leal da Costa Herbarium (ALCB), located at the Institute of Biology of the Federal University of Bahia (Salvador, BA, Brazil), under number 106742.

The compound CV2 was provided by Prof. Lucas Abreu. The extraction and isolation of CV2 were performed according to the methodology previously described.^8^ Briefly, *C. velutinus* roots were oven-dried at 32 °C for 72 h, then ground and macerated with organic solvents. The dried and powdered material was macerated with hexane and methanol (MeOH). The hexane extract obtained was subjected to column chromatography (CC) using silica gel as the stationary phase. Hexane and ethyl acetate solvents, either pure or in binary mixtures, were used as the mobile phase. The fractions obtained were analyzed by thin layer chromatography (TLC) and subjected to nuclear magnetic resonance (NMR) analysis. The purity level of CV2 (99%) was evaluated based on an HPLC chromatogram (Figure S1 Supporting Information) and was stored in a freezer at −80 °C to prevent degradation and to maintain the reproducibility of the tests.

### 4.5 Assessment of CV2 Cytotoxicity and Calculation of the Selectivity Index

CV2 cytotoxicity was assessed using the 3-(4,5-dimethylthiazol-2-yl)-2,5-diphenyltetrazolium (MTT) assay.^117^ Cells were seeded in 96-well plates (Guangzhou Jet Bio-Filtration Co., Ltd., Guangzhou, China) at a density of 3 × 10^5^ cells/mL (MCF-7, HCT-116, MDA-MB-231, HeLa, SK-MEL-128, or MCF-10A) or 1 × 10^6^ cells/mL (PBMCs) and incubated at 37 °C with 5% CO_2_. After 24 h, the cells were treated with 100 μL of CV2 (3.12–200 μM for human tumor cell lines and MCF-10A; 1.25–20 μM for PBMCs) or doxorubicin (DXR) (0.15–5 μM), which was used as the standard drug. The negative control group received culture medium containing the vehicle (DMSO) only. The final DMSO concentration did not exceed 0.1% (v/v), and control cells were exposed to the same vehicle concentration used in the treated groups. After the incubation period (24, 48, or 72 h, as applicable), the supernatant (110 μL) was removed and MTT solution (10 μL, 5 mg/mL in PBS) was added and incubated (5% CO2, 37 °C) for 4 h. The precipitated formazan crystals were dissolved with sodium dodecyl sulfate (SDS) (100 μL/well) overnight^43,44,116^ and the optical density was measured using a microplate reader (Synergy HT, BioTek, Winooski, VT, USA) at λ = 570 nm and is used to estimate the IC_50_ (half maximal inhibitory concentration) values. The selectivity index (SI) was calculated as the ratio between the IC_50_ of the nontumor cell line (MCF-10A) and the IC_50_ of the tumor cell lines (MCF-7, MDA-MB-231, HCT-116, HeLa, SK-MEL-128). Three independent experiments were performed in triplicate.

### 4.6 Cell Cycle Analysis

To evaluate the interference of CV2 on cell cycle progression, MCF-7 cells were seeded in 24-well plates at a density of 1 × 10^5^ cells/per well and incubated at 37 °C in a humidified atmosphere containing 5% CO_2_. After 24 h, cells were treated with CV2 at concentrations of 5 or 10 μM (corresponding to the IC_50_ and twice the IC_50_ value, respectively) or doxorubicin (DXR; 2 μM, corresponding to the IC_50_) for 48 h. Following treatment, cells were detached using 0.25% trypsin-EDTA at 37 °C for 3–5 min, and the reaction was stopped by adding complete culture medium supplemented with 10% fetal bovine serum (FBS). Cells were collected by centrifugation (500 × g, 20 °C, 5 min), washed with phosphate-buffered saline (PBS), fixed in ice-cold 70% ethanol, and stored at −20 °C until analysis. For cell analysis, cells were recovered by centrifugation (400 × g, 4 °C, 10 min), permeabilized with 1% Triton X-100 for 30 min at room temperature, washed with PBS, and incubated with RNase (0.1 mg/mL) and propidium iodide (PI; 0.05 mg/mL) in the dark for 30 min. Samples were analyzed using a BD FACSCanto II flow cytometer (BD Biosciences, Woburn, MA, USA), and 10,000 events were acquired per sample. Data acquisition was performed using BD FACSDiva 6.0 software. Cell populations were initially gated based on forward scatter (FSC) and side scatter (SSC) parameters to exclude debris, and doublets were excluded using width-versus-area discrimination. DNA content was determined by PI fluorescence, allowing quantification of the percentage of cells in each phase of the cell cycle as well as the sub-G1 population.^44,116^ Three independent experiments were performed in triplicate.

### 4.7 Apoptosis Evaluation

Apoptosis was assessed by Annexin V–fluorescein isothiocyanate (FITC) and propidium iodide (PI) double staining followed by flow cytometry. MCF-7 cells were seeded in 24-well plates at a density of 1 × 10^5^ cells per well and incubated at 37 °C in a humidified atmosphere containing 5% CO_2_. After 24 h, cells were treated with CV2 (5 or 10 μM) or doxorubicin (DXR; 2 μM) for 48 h.

Following treatment, cells were detached using 0.25% trypsin-EDTA at 37 °C for 3–5 min, and the reaction was stopped by the addition of complete culture medium supplemented with 10% fetal bovine serum (FBS). Cells were collected by centrifugation (500 × g, 20 °C, 5 min), resuspended in binding buffer, and incubated on ice in the dark for 10 min with 5 μL of Annexin V-FITC and 10 μL of propidium iodide (20 μg/mL),^10,116^ according to the manufacturer’s instructions (eBioscience, Thermo Fisher Scientific, Rochester, NY, USA).

Samples were analyzed using a BD FACSCanto II flow cytometer (BD Biosciences, Woburn, MA, USA), and 10,000 events were acquired per sample. Data acquisition was performed using BD FACSDiva 6.0 software. Cell populations were initially gated based on forward scatter (FSC) and side scatter (SSC) parameters to exclude debris, and doublets were excluded using width-versus-area discrimination. Three independent experiments were performed in triplicate.

### 4.8 Evaluation of Reactive Oxygen Species (ROS) Involvement in the CV2 Antitumor Effect

#### 4.8.1 Quantification of ROS Using the 2,7-Dichlorodihydrofluorescein Diacetate (DCFH-DA) Assay

ROS levels were quantified using the 2′,7′-dichlorodihydrofluorescein diacetate (DCFH-DA) assay, as previously described.^35^ DCFH-DA is a nonfluorescent, cell-permeable probe that is hydrolyzed by intracellular esterases to 2′,7′-dichlorodihydrofluorescein (DCFH), which is subsequently oxidized by ROS to the highly fluorescent compound 2′,7′-dichlorofluorescein (DCF).

MCF-7 cells were seeded in 24-well plates at a density of 1 × 10^5^ cells/mL (1000 μL per well) and incubated for 24 h at 37 °C in a humidified atmosphere containing 5% CO_2_. Cells were then treated with CV2 (5 or 10 μM), doxorubicin (DXR; 2 μM), or hydrogen peroxide (H_2_O_2_; 500 μM) for 30 min or 3 h. Following treatment, cells were detached using 0.25% trypsin-EDTA at 37 °C for 3–5 min, and the reaction was stopped by the addition of complete culture medium supplemented with 10% fetal bovine serum (FBS). Cells were collected by centrifugation (500 × g, 20 °C, 5 min), resuspended in phosphate-buffered saline (PBS), and incubated with DCFH-DA (10 μM) for 30 min at 37 °C in the dark.^43^

The percentage of DCF-positive cells was determined using a BD FACSCanto II flow cytometer (BD Biosciences, Woburn, MA, USA), acquiring 10,000 events per sample. Data acquisition was performed using BD FACSDiva 6.0 software. Cell populations were initially gated based on forward scatter (FSC) and side scatter (SSC) parameters to exclude debris, and doublets were excluded using width-versus-area discrimination. Three independent experiments were performed in triplicate for each treatment period (30 min and 3 h).

#### 4.8.2 Assessment of CV2 Cytotoxicity in the Absence or Presence of N-Acetylcysteine (NAC)

To investigate the involvement of reactive oxygen species (ROS) in CV2-induced cytotoxicity, MCF-7 cells were seeded in 96-well plates at a density of 1 × 10^5^ cells/mL (100 μL per well) and incubated for 24 h at 37 °C in a humidified atmosphere containing 5% CO_2_. Cells were then pretreated in the presence or absence of N-acetylcysteine (NAC; 5 mM) for 3 h under the same incubation conditions.

Following pretreatment, cells were exposed to CV2 (5 or 10 μM; 100 μL per well) for 72 h. After treatment, 110 μL of the culture medium was removed, and MTT solution (10 μL, 5 mg/mL) was added. Cells were further incubated for 4 h at 37 °C in a 5% CO_2_ atmosphere. Formazan crystals were subsequently dissolved in sodium dodecyl sulfate (SDS; 100 μL per well) overnight, and cell viability (%) was determined by measuring absorbance at 570 nm using a Synergy HT microplate reader (BioTek, Winooski, VT, USA).^43,118^ Three independent experiments were performed in triplicate.

### 4.9 Assessment of CV2 Cytotoxicity in the Absence or Presence of MAPK Inhibitors (iMAPKs)

To investigate the involvement of mitogen-activated protein kinase (MAPK) pathways in CV2-induced cytotoxicity, the MTT assay was performed in the presence or absence of specific MAPK inhibitors. MCF-7 cells were seeded in 96-well plates at a density of 1 × 10^5^ cells/mL and incubated for 24 h at 37 °C in a humidified atmosphere containing 5% CO_2_. Cells were then pretreated for 3 h with the extracellular signal-regulated kinase 1/2 (ERK1/2) inhibitor U0126 (5 μM), the c-Jun N-terminal kinase (JNK) inhibitor SP600125 (5 μM), or the p38 MAPK inhibitor PD169316 (5 μM). All inhibitors were obtained from Sigma-Aldrich (St. Louis, MO, USA). Following pretreatment, cells were exposed to CV2 (5 or 10 μM) for 72 h under the same incubation conditions.

After treatment, 110 μL of the culture medium was removed, and MTT solution (10 μL, 5 mg/mL) was added. Cells were further incubated for 4 h at 37 °C in a 5% CO_2_ atmosphere. Formazan crystals were subsequently dissolved in sodium dodecyl sulfate (SDS; 100 μL per well) overnight, and cell viability (%) was determined by measuring absorbance at 570 nm using a Synergy HT microplate reader (BioTek, Winooski, VT, USA).^10,118^ Three independent experiments were performed in triplicate.

### 4.10 Investigation of MAPK Signaling by Flow Cytometry

To investigate the effects of CV2 on mitogen-activated protein kinase (MAPK) signaling, MCF-7 cells were seeded in 24-well plates at a density of 1 × 10^5^ cells/mL (1000 μL per well) and incubated for 24 h at 37 °C in a humidified atmosphere containing 5% CO_2_. Cells were then treated with CV2 (5 or 10 μM, corresponding approximately to the IC_50_ and twice the IC_50_ value, respectively) or doxorubicin (DXR; 2 μM, corresponding to the IC_50_) for 48 h.

Following treatment, cells were detached using 0.25% trypsin-EDTA at 37 °C for 3–5 min, and the reaction was stopped by the addition of complete culture medium supplemented with 10% fetal bovine serum (FBS). Cells were collected by centrifugation (500 × g, 20 °C, 5 min), fixed in binding buffer containing 4.2% formaldehyde for 10 min at 37 °C, and permeabilized with 87.68% methanol (w/w). Cells were subsequently recovered by centrifugation (250 × g, 4 °C, 10 min) and stained with antiphospho-ERK1/2 (clone 20A, 20 μL/test), antiphospho-JNK1 (clone N9–66, 5 μL/test), or antiphospho-p38 MAPK antibodies (clone 36/p38, 20 μL/test) (BD Biosciences, Franklin Lakes, NJ, USA). The final staining volume was adjusted to 100 μL, according to the manufacturer’s instructions. After incubation with the antibodies for 30 min at room temperature in the dark, cells were washed, centrifuged (300 × g, 4 °C, 5 min), and resuspended in buffer for flow cytometric analysis.^10,118^

Samples were analyzed using a BD FACSCanto II flow cytometer (BD Biosciences, Woburn, MA, USA), and 10,000 events were acquired per sample. Data acquisition was performed using BD FACSDiva 6.0 software. Cell populations were initially gated based on forward scatter (FSC) and side scatter (SSC) parameters to exclude debris, and doublets were excluded using width-versus-area discrimination. Three independent experiments were performed in duplicate.

### 4.11 Analysis of NF-κB and PKB/AKT Signaling by Flow Cytometry

To investigate the effects of CV2 on Nuclear Factor kappa B (NF-κB) and Protein Kinase B (PKB/AKT) signaling pathways, MCF-7 cells were seeded in 24-well plates at a density of 1 × 10^5^ cells/mL (1000 μL per well) and incubated for 24 h at 37 °C in a humidified atmosphere containing 5% CO_2_. Cells were then treated with CV2 (5 or 10 μM, corresponding approximately to the IC_50_ and twice the IC_50_ value, respectively) or doxorubicin (DXR; 2 μM, corresponding to the IC_50_) for 48 h.

Following treatment, cells were detached using 0.25% trypsin-EDTA at 37 °C for 3–5 min, and the reaction was stopped by the addition of complete culture medium supplemented with 10% fetal bovine serum (FBS). Cells were collected by centrifugation (500 × g, 20 °C, 5 min), fixed in binding buffer containing 4.2% formaldehyde for 10 min at 37 °C, and permeabilized with 87.68% methanol (w/w) for 30 min at 4 °C. Cells were subsequently recovered by centrifugation (250 × g, 4 °C, 10 min) and stained with antiphospho-NF-κB p65 (clone K10–895.12.50, 20 μL/test) or antiphospho-PKB/AKT antibodies (clone M89-61, 20 μL/test) (BD Biosciences, Franklin Lakes, NJ, USA). The final staining volume was adjusted to 100 μL, according to the manufacturer’s instructions. After incubation with the antibodies for 30 min at room temperature in the dark, cells were washed, centrifuged (300 × g, 4 °C, 5 min), and resuspended in buffer for flow cytometric analysis.

Samples were analyzed using a BD FACSCanto II flow cytometer (BD Biosciences, Woburn, MA, USA), and 10,000 events were acquired per sample. Data acquisition was performed using BD FACSDiva 6.0 software. Cell populations were initially gated based on forward scatter (FSC) and side scatter (SSC) parameters to exclude debris, and doublets were excluded using width-versus-area discrimination.^10^ Three independent experiments were performed in duplicate.

### 4.12 Zebrafish Embryos

Zebrafish embryos (AB strain) approximately 1 h postfertilization (hpf) were obtained from UniPOM/UFPB. The breeders were maintained in a controlled recirculating system with water quality monitoring, temperature (26 ± 1 °C), and a light/dark cycle. On the day prior to the experiment, adult fish (2:1 male-to-female ratio) were placed in breeding tanks for embryo collection. Only embryos with ≥90% fertilization and no abnormalities were selected using an inverted microscope. The experiment was approved by the UFPB Ethics Committee (protocol no. 8881290419).

Zebrafish embryos were used as a vertebrate model for early toxicological screening because they enable rapid assessment of lethality and developmental toxicity in a whole-organism system and are widely employed in accordance with OECD Guideline No. 236.

### 4.13 Investigation of the Acute Toxicity of CV2 in Zebrafish Embryos

The Fish Embryo Toxicity (FET) test was conducted independently with CV2 according to OECD guideline No. 236^106^. Embryos up to 3 hpf were exposed by immersion under static conditions to five increasing concentrations of CV2 (2.0–4.0 μM) for 96 h in 96-well plates. For each concentration tested, 20 fertilized embryos (one embryo per well) were evaluated. Four embryos exposed only to E3 medium were included as internal controls for each concentration tested. Additional negative (E3 medium) and solvent (0.1% DMSO) controls were also included. The volume of test solution in each well was 0.3 mL. The plates were covered with their lids to minimize evaporation and maintained in a humidified chamber at 26 ± 1 °C throughout the exposure period.

Embryos were monitored daily for 96 h. Lethality was assessed according to OECD Guideline No. 236 based on the following end points: egg coagulation, lack of somite formation, lack of detachment of the tail-bud from the yolk sac, and absence of heartbeat. Embryos presenting any of these end points were considered dead.

Nonlethal developmental abnormalities were also evaluated, including eye malformation, otolith malformation, mouth malformation, spine malformation, changes in body pigmentation, hatching delay, yolk sac edema, yolk sac deformation, pericardial edema, head edema, blood clotting, and reduced body size. Observations were performed using a stereomicroscope and documented throughout the experiment.

After 96 h of exposure, concentration–response data were used to determine the lethal concentration for 50% of the embryos (LC_50_) and its corresponding 95% confidence intervals.^43,116,119^

### 4.14 Evaluation of Oxidative Stress Biomarkers in Zebrafish Larvae

To investigate the effects of CV2 on antioxidant defense systems, the Fish Embryo Toxicity (FET) assay was repeated under the same experimental conditions described above. Embryos were exposed to sublethal concentrations of CV2 (0.5, 1, and 2 μM) for 96 h. At the end of the exposure period, surviving larvae were rapidly frozen in 0.1 M sodium phosphate buffer (pH 7.4).

Larvae were then homogenized in ice-cold 0.9% NaCl solution (1:9, w/v). The homogenates were centrifuged at 10,000 × g for 10 min at 4 °C, and the resulting supernatants were used for biochemical analyses. Soluble protein content was determined for normalization of enzymatic activity.

The activities of glutathione S-transferase (GST), glutathione peroxidase (GPx), and catalase (CAT) were determined according to Domingues et al. (2010).^120^ All assays were performed in quadruplicate for each enzyme, and enzyme activities were expressed relative to total protein content.

### 4.15 Statistical Analysis

Cell viability data were analyzed by nonlinear regression using a four-parameter logistic (4PL) sigmoidal dose–response model with log-transformed drug concentrations. IC_50_ values were estimated from concentration–response curves generated from serially diluted concentrations spanning the dynamic range of the biological response and are presented with their corresponding 95% confidence intervals (95% CI). The goodness-of-fit of the nonlinear regression models was assessed by the coefficient of determination (R^2^), and all fitted curves showed R^2^ values greater than 0.9806. Results are expressed as mean ± standard error of the mean (SEM) from at least three independent biological experiments.

Statistical analysis were performed using one-way analysis of variance (ANOVA) followed by Tukey’s post hoc test for multiple comparisons. Differences were considered statistically significant when *p* < 0.05. For the zebrafish embryotoxicity assay, the median lethal concentration (LC_50_) and its corresponding 95% confidence interval (95% CI) were determined by probit regression analysis.

## 5 Conclusions

This study provides the first experimental insights into the mechanisms underlying the antitumor activity of (E)-4-(1-epoxy-7,8-propen)­phenyl benzoate (CV2), a phenylpropanoid isolated from *Croton velutinus*. CV2 exhibited potent cytotoxic activity against MCF-7 breast cancer cells and induced apoptosis through mechanisms associated with intracellular ROS accumulation and modulation of signaling pathways involved in cell fate regulation. Functional assays demonstrated that oxidative stress, as well as JNK and p38 MAPK signaling, play central roles in the cytotoxic response elicited by CV2. In addition, the modulation of AKT and NF-κB highlights the complexity of the cellular stress-response networks triggered by this compound. Toxicity assessments in PBMCs and zebrafish embryos further demonstrated that CV2 possesses relevant biological activity in nontumor models, supporting the need for additional studies to better define its therapeutic window. Collectively, these findings significantly expand the current knowledge of CV2 pharmacology, establish the first mechanistic framework for its antitumor activity, and support its continued investigation as a promising lead compound for breast cancer drug development.

## Supplementary Material



## Notes

An HPLC chromatogram (PDF)
