# Supplementary material for: Signaling Pathways Mediating the Cytotoxicity of (E)-4-(1-Epoxy-7,8-propenyl)phenyl Benzoate, a Phenylpropanoid from Croton velutinus, in MCF‑7 Cells
Source: ACS Omega. 2026 Oct 2;11(40):60877–96. doi: 10.1021/acsomega.6c04220 (PMC13670846; doi:10.1021/acsomega.6c04220)
Supplement: Supplementary file 1 [file ao6c04220_si_001.pdf]

Chromatogram

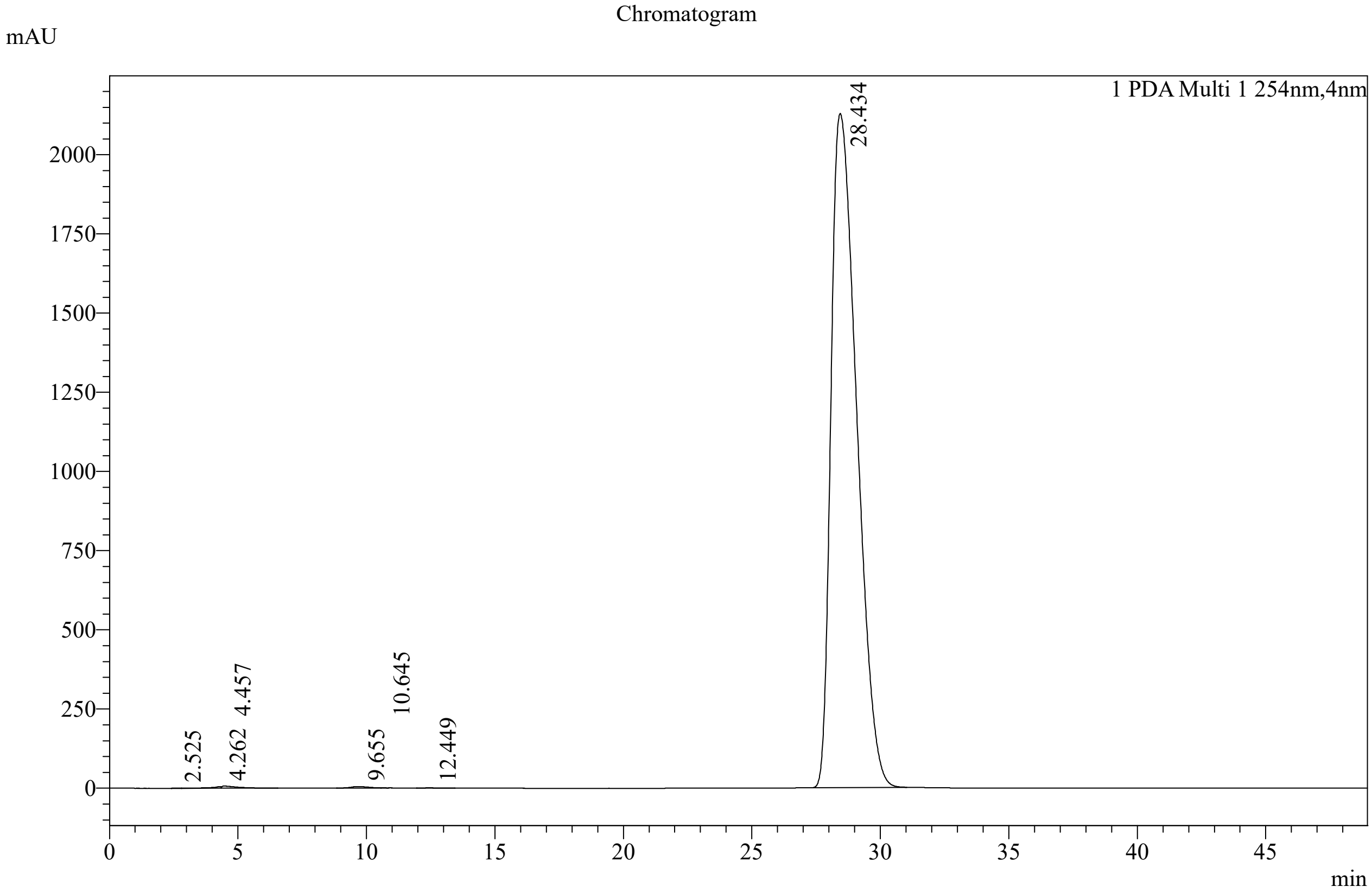


Peak Table

PDA Ch1 254nm

| Peak# | Ret. Time | Area | Area% |
|---|---|---|---|
| 1 | 2.525 | 5889 | 0.004 |
| 2 | 4.262 | 144429 | 0.097 |
| 3 | 4.457 | 296855 | 0.200 |
| 4 | 9.655 | 204362 | 0.137 |
| 5 | 10.645 | 1654 | 0.001 |
| 6 | 12.449 | 45312 | 0.030 |
| 7 | 28.434 | 148008658 | 99.530 |
| Total | | 148707159 | 100.000 |
